# Nuclear Localization Signals for Optimization of Genetically Encoded Tools in Neurons

**DOI:** 10.3389/fcell.2022.931237

**Published:** 2022-07-19

**Authors:** Maksim M. Karasev, Mikhail Baloban, Vladislav V. Verkhusha, Daria M. Shcherbakova

**Affiliations:** ^1^ Medicum, Faculty of Medicine, University of Helsinki, Helsinki, Finland; ^2^ Department of Genetics and Gruss-Lipper Biophotonics Center, Albert Einstein College of Medicine, Bronx, NY, United States

**Keywords:** nuclear localization signal, nuclear transport, importins, neurons, optogenetic tools, near-infrared

## Abstract

Nuclear transport in neurons differs from that in non-neuronal cells. Here we developed a non-opsin optogenetic tool (OT) for the nuclear export of a protein of interest induced by near-infrared (NIR) light. In darkness, nuclear import reverses the OT action. We used this tool for comparative analysis of nuclear transport dynamics mediated by nuclear localization signals (NLSs) with different importin specificities. We found that widely used KPNA2-binding NLSs, such as Myc and SV40, are suboptimal in neurons. We identified uncommon NLSs mediating fast nuclear import and demonstrated that the performance of the OT for nuclear export can be adjusted by varying NLSs. Using these NLSs, we optimized the NIR OT for light-controlled gene expression for lower background and higher contrast in neurons. The selected NLSs binding importins abundant in neurons could improve performance of genetically encoded tools in these cells, including OTs and gene-editing tools.

## Introduction

Neurons are highly specialized cells that perform integration of input signals at individual synapses and require communication between distant parts of the cell with its soma containing the nucleus. Therefore, nuclear transport is also specialized. In neurons, importins transport proteins from synapses along axons and dendrites to the nucleus, in addition to active nuclear import in the soma (Lever et al., 2015).

Optogenetic tools (OTs) allow control of cellular processes at a precise time and spatial location defined by instigators. Non-opsin OTs are used for light-induced protein-protein interactions, protein degradation, and activation of signaling cascades, etc. (Ma et al., 2017; Rost et al., 2017; Varady and Distel, 2020). The functionality of some of these tools depends on nuclear transport and, therefore, may vary in different cell types.

Interaction of nuclear localization signals (NLSs) with nuclear transport receptors, karyopherins, is required for active nuclear transport (Cautain et al., 2015; Eibauer et al., 2015). The karyopherin protein family consists of two subfamilies: karyopherin-*α* (KPNA, importins) and karyopherin-*β* (KPNB, importins, exportins, and bidirectional transporters) (Cautain et al., 2015). The human genome encodes seven KPNA isoforms, which are classified into clades or families *α*1, *α*2, and *α*3 (Goldfarb et al., 2004; Kelley et al., 2010). KPNA isoforms demonstrate substrate preference (Köhler et al., 1999; Pumroy and Cingolani, 2015; Kimura et al., 2017), however, there are functional redundancies in cargo binding specificity (Ushijima et al., 2005; Friedrich et al., 2006; Mackmull et al., 2017). The expression of karyopherins varies across different cell and tissue types, and developmental stages (Quan et al., 2008; Pumroy and Cingolani, 2015).

Classical NLSs, such as SV40 large T antigen NLS and Myc NLS, bind KPNA leading to a formation of a complex KPNB1-KPNA-[NLS-cargo], which translocates through the nuclear pore to the nucleus (Cautain et al., 2015). Non-classical NLSs bind KPNB directly without interacting with KPNA (Chook and Süel, 2011). The most studied example of them is the so-called PY-NLS group that is recognized by TNPO1 (Chook and Süel, 2011).

Several factors influence nuclear import: NLS affinity (Efthymiadis et al., 1997; Xiao et al., 1998; Hodel et al., 2006; Timney et al., 2006; Yang et al., 2010), amino acids surrounding NLS (Xiao et al., 1998; Friedrich et al., 2006), protein phosphorylation (Nardozzi et al., 2010), and protein cargo size (Hodel et al., 2006; Böhm et al., 2017). NLS affinity directly correlates with the cargo distribution between the nucleus and the cytoplasm (nucleocytoplasmic ratio) in steady-state, as well as with the rate of nuclear import.

Several non-opsin OTs utilize light control of nuclear localization to regulate the activity of proteins by keeping them inaccessible in the dark and releasing them under illumination (Di Ventura and Kuhlman, 2016). These tools use different light-sensing protein modules. Red-light (660 nm) controlled heterodimeric protein-protein interaction between plant PhyB photoreceptor and its interacting protein PIF were adapted for light-induced nuclear translocation of a protein of interest (Beyer et al., 2015; Juillot et al., 2016; Noda and Ozawa, 2018). The limitations of PhyB-PIF are a requirement to supply exogenous chromophore phycocyanobilin and high light sensitivity to dim ambient light complicating system handling (Pathak et al., 2014).

Several single-component blue-light sensing OTs for nuclear transport were engineered based on the AsLOV2 domain from *Avena sativa* phototropin 1. In these tools, an NLS or a nuclear export signal (NES) was incorporated into the C-terminal Jα helix of AsLOV2 to make them inaccessible in the darkness and “uncaged” under illumination when the Jα helix is undocked and unfolded (Niopek et al., 2014, 2016; Yumerefendi et al., 2015, 2016). Example applications of these OTs include light control of mitosis initiation (Niopek et al., 2014), gene expression (Niopek et al., 2014, 2016; Yumerefendi et al., 2015, 2016), light control of CRM1 binding NES to prevent nuclear export of cargoes (Niopek et al., 2016). *As*LOV2 provides advantages of relatively small size (app. 16 kDa) and usage of flavin chromophores that is ubiquitously present in many cell types. The main limitation of the *As*LOV2-based systems is the requirement for blue light. In addition to poor penetration in tissues (Rumyantsev et al., 2016), blue light is toxic (Marek et al., 2019) and can alter neurons morphology (Diaz Vera et al., 2021) and physiology by activating the expression of immediate-early genes (Tyssowski and Gray, 2019).

Near-infrared (NIR) light above 700 nm is minimally toxic, penetrates deep in tissue (Rumyantsev et al., 2016), and does not influence the behavior of small animals, which cannot see it (Peirson et al., 2018). As a light-sensing module, NIR OTs use bacterial phytochrome photoreceptors incorporating endogenously available chromophore biliverdin (BV). Bacterial phytochrome BphP1 from *Rhodopseudomonas palustris* senses 720–780 nm. In the darkness, BphP1 adopts the Pfr state (inactive) with the absorbance maximum at 760 nm. Upon illumination, it undergoes photoconversion into the Pr state (active) with the absorbance maximum at 680 nm. In the Pr state, it binds PpsR2 (Kaberniuk et al., 2016) or its small engineered derivative QPAS1 (Redchuk et al., 2017). BphP1 was used in several OTs for the regulation of transcription (Kaberniuk et al., 2016; Redchuk et al., 2017, 2018b, 2018a), intracellular protein targeting (Redchuk et al., 2017; 2018b; 2018a, 2020), and cell signaling control (Kaberniuk et al., 2016; Redchuk et al., 2020). Non-invasive control of bacteriophytochome-based OTs was demonstrated in living mice (Kaberniuk et al., 2016; Shao et al., 2017; Fomicheva et al., 2019; Wu et al., 2020; Wang et al., 2021; Yu et al., 2022). The functionality of light-induced BphP1-QPAS1 interaction was also shown in primary neurons (Redchuk et al., 2018b).

In this study, we developed a NIR light-controlled OT for nuclear export of a protein of interest. Using this OT, we identified a difference in nuclear transport dynamics for widely used Myc NLS in primary cortical neurons and non-neuronal cells. Therefore, we further applied this OT to study the nuclear transport dynamics of uncommon NLSs with various importin specificities. We found NLSs that allow fast nuclear import in neurons and showed that the performance of the NIR OT for nuclear export can be adjusted. Using selected NLSs, we optimized a NIR light-activated gene expression system for lower background and higher contrast in neurons.

## Results

### Development of an OT for NIR Light-Controlled Nuclear Export

**FIGURE 1:**
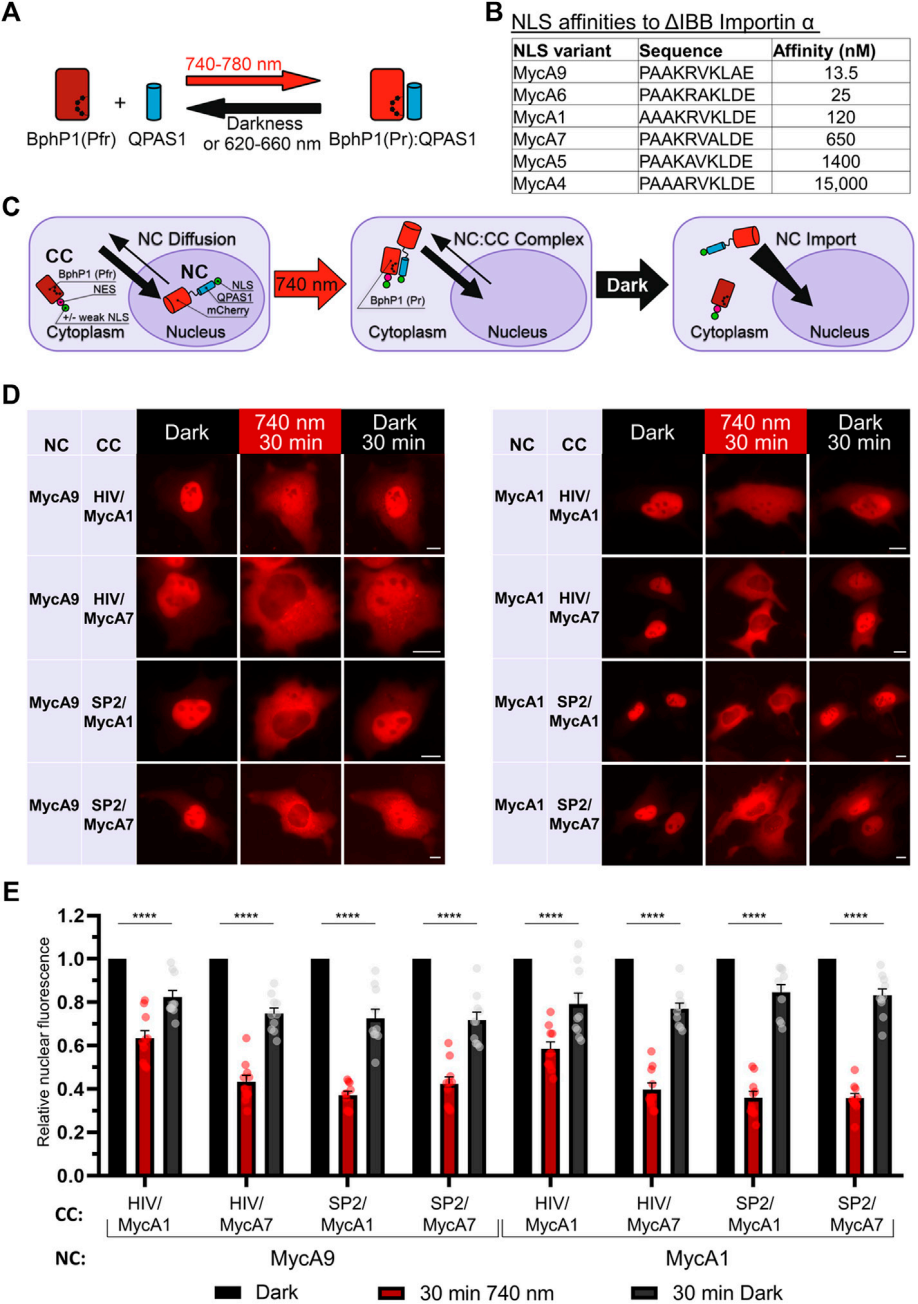
Development of a NIR OT for light-controlled nuclear export. **(A)** Schematic representation of the BphP1-QPAS1 NIR light-induced interaction. **(B)** A selected range of Myc NLS mutants with measured affinities to importin *a* with deleted importin *ß*-binding domain (ΔIBB) (Hodel et al., 2001) (*n* = 10). **(C)** Model of interaction for the NIR OT design with the QPAS1-based nuclear component (NC) and the BphP1-based cytoplasmic component (CC). In darkness, the NC is sequestered in the nucleus with its negligible amount diffusing to the cytoplasm. Under illumination, it re-localizes to the cytoplasm through interaction with the CC that contains strong NES. In darkness, the NC:CC complex dissociates, and the NC is imported back to the nucleus. **(D)** Representative images of the mCherry-labeled NC obtained with widefield microscopy in living HeLa cells. Points before illumination, after 30 min of 740 nm light illumination, and after subsequent 30 min in darkness are shown. Scale bar, 10 μm. **(E)** Quantification of relative nuclear fluorescence of the NC for the NC-CC combinations displayed on **(D)**. Mean values for individual cells ± S.E.M. were calculated (*n* = 10). Statistical significance was determined using one-way ANOVA and Tukey’s test. ****p < 0.0001. SP2—Super-PKI-2 NES; HIV—HIV-1 Rev NES.

As a light sensing module, we used a BphP1-QPAS1 pair interacting under 720–780 nm illumination (Figure 1A). We screened NLS and NES combinations in the two protein fusions corresponding to the nuclear component (NC) carrying a cargo protein of interest and the cytoplasmic component (CC) in 293T and HeLa cells. As a model cargo, we used mCherry fused with M13 calmodulin-binding peptide and a fragment of split tobacco etch virus protease. We tested a set of Myc NLS mutants that differed in affinity to importin *a* (Hodel et al., 2001) (Figure 1B) in combination with NES signals: strong Super-PKI-2 NES (Güttler et al., 2010) and weaker NES of HIV-1 Rev (Fischer et al., 1995).

Initially, we aimed to develop the CC that would shuttle between the nucleus and the cytoplasm and pull the NC to the cytoplasm upon illumination. We tried both members of the BphP1-QPAS1 optogenetic pair as the NC and the CC. Bulkier BphP1 (80 kDa) fused to cargo as the NC (102 kDa) should not diffuse to the cytoplasm (Supplementary Figure S1A). We identified a combination that resulted in partial relocalization of the BphP1-containing NC to the cytoplasm under light (Supplementary Figure S1B). The working QPAS1-based CC variants contained weaker MycA7 and MycA5 NLSs. Still, this relocalization was inefficient (Supplementary Figure S1C). We did not observe any relocalization with the CC variant with no NLS, which indicates that shuttling of the QPAS1-based CC component is critical.

Alternatively, we used the BphP1-based CC and the NC containing QPAS1 fused to NLS and the cargo (Figure 1C). In this setup, we used strong NES and weak or no NLS on the large BphP1-containing CC (86 kDa) and the strong NLSs on the smaller diffusing NC. We found that the NC size at the border of the diffusion limit (67 kDa) enabled both 1) its sequestration in the nucleus and 2) its diffusion to the cytoplasm. Under illumination, the NC efficiently re-localized to the cytoplasm (Figures 1D,E) through interaction with the BphP1-containing CC. The NC variants containing MycA9 and weaker MycA1 NLSs relocalized similarly (Figure 1E) in combination with the CC with strong Super-PKI2 NES. We found that removing NLS entirely from the CC did not influence the performance (Supplementary Figure S2). This indicates that the diffusion of the NC, not the shuttling of the CC, is the main mechanism behind the light-induced NC relocalization (Figure 1C). In darkness, BphP1 released the QPAS1-containing NC that returned to the nucleus. The final OT contained QPAS1 fused with the cargo and a strong MycA9 NLS as the NC and BphP1 fused with a strong Super-PKI2 NES with (or without) attached weak MycA1 NLS as the CC.

### Nuclear Transport Dynamics in Neuronal and Non-neuronal Cells

**FIGURE 2:**
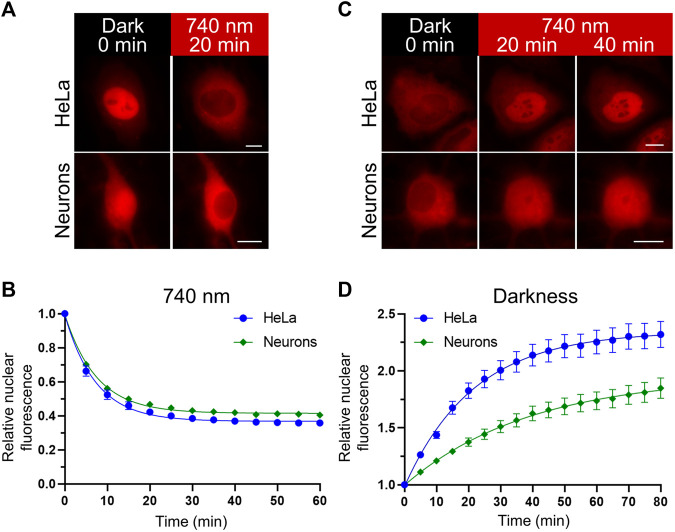
Nuclear transport dynamics in non-neuronal cells and neurons. **(A,C)** Representative images showing relocalization of the mCheery-labeled NC in HeLa and primary rat cortical neurons (at days *in vitro* (DIV) 11) upon NIR (740 nm) light illumination **(A)** and subsequent darkness **(C)** captured with widefield fluorescence microscopy in living cells. Scale bar, 10 μm. **(B,D)** Quantification of the NC relocalization dynamics corresponding to **(A,C)** respectively. Mean values ± S.E.M. were calculated for individual cells (*n* = 20 each).

We compared the kinetics of the nuclear transport obtained with the NIR OT in HeLa and primary rat cortical neuronal cultures (Figure 2). We detected relocalization of the mCherry-labelled NC in live cells. Upon illumination, the NC was efficiently exported in both cell types. The estimated export half-times (*τ*_1/2_) for HeLa and neurons were 5.05 and 5.18 min, respectively (Figures 2A,B). Then we compared the kinetics of nuclear import in darkness. We observed a significant difference in the NC nuclear import rates between HeLa and neurons (Figures 2C,D). The estimated import half-time for primary neurons was 27.28 min, which is almost twice higher than for HeLa (*τ*_1/2_ = 15.05 min). Besides the nuclear import, these measured half-times include the relatively short time needed for QPAS1 release from BphP1 [the BphP1-QPAS1 dissociation was measured as ∼4.4 min in darkness (Redchuk et al., 2017)].

### Selection of Uncommon NLSs Mediating Fast Nuclear Transport in Neurons

**FIGURE 3:**
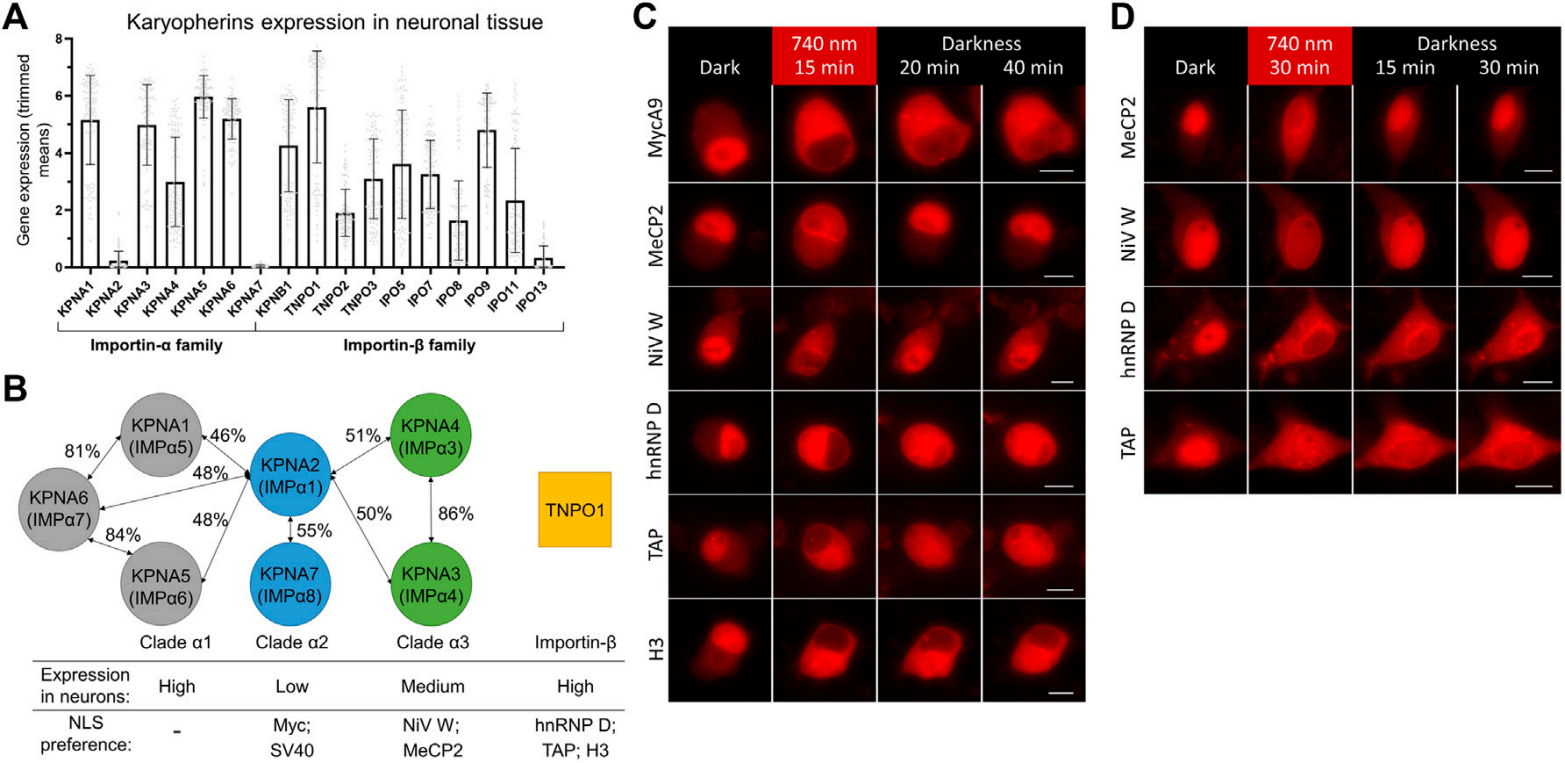
Screening of NLSs with different importin specificities to improve nuclear import dynamics in neurons. **(A)** Neuronal tissue transcriptome profiling for karyopherins built using Human Multiple Cortical Areas SMART-seq dataset (Hodge et al., 2019). Data for neuronal cells are grouped by importin gene. Bars represent mean gene expression values (±SD) for all cortical neuronal sub-types determined by single cell gene expression profiling. **(B)** Schematics of three clades of human importin *a* proteins and TNPO1 transporter from the importin *ß* family. Percentages of protein homology between importin *a* isoforms are specified (Goldfarb et al., 2004; Kelley et al., 2010). Relative importin expression levels and selected NLSs specificities are indicated. **(C,D)** Representative fluorescent images of the mCherry-labeled NC in living N2a cells **(C)** and cultured primary neurons at DIV 10 **(D)** expressing the NIR OT for light-controlled nuclear export with different NLSs. Points before illumination and after 15 min of 740 nm light illumination at indicated time points are shown. Scale bar, 10 μm.

To find a reason for a difference in nuclear import kinetics, we turned to available data on expression levels of importin *a* isoforms in neuronal and non-neuronal cells. Transcriptome analysis of neuronal tissue showed a strikingly low level of transcripts for two importins forming clade *α*2: KPNA2 and KPNA7 (Hodge et al., 2019) in cortical neurons (Figure 3A). Lower or absent expression of KPNA2 in mice neural tissue also was shown in an earlier study (Hosokawa et al., 2008). At the same time, the level of KPNA2 in baseline transcription profiling data for HeLa was relatively high (Bekker-Jensen et al., 2017) (Supplementary Figure S3).

It was shown that SV40 and Myc NLSs bind preferentially KPNA2, less strongly KPNA1, and only weakly KPNA4 (Miyamoto et al., 1997; Nadler et al., 1997) and KNPA3 (Nachury et al., 1998). Interestingly, peptide library screening showed that optimal sequences for binding to KPNA2 are very close to SV40 NLS (Yang et al., 2010). KPNA2 preference and its strikingly low expression level in neurons suggest an explanation for a slower import of Myc NLS, and possibly related SV40 NLS, compared to non-neuronal cells.

To find alternative NLSs that mediate fast and efficient nuclear transport, we decided to test NLSs that have importin preferences other than KPNA2. We searched the literature for short high-affinity NLSs, which have been shown to work in fusion with heterologous proteins. We selected five NLSs preferably binding KPNA3, KPNA4, or TNPO1 (Figure 3B). First, the NLS of W protein of Nipah virus (NiV W) preferentially binds KPNA4 and KPNA3 with high affinity (K_D_ is 14.4 nM for KPNA4) (Smith et al., 2018). Second, the NLS identified as a 249–272 region of methyl-CpG binding protein 2 (MeCP2) primarily binds KPNA3 and KPNA4 (Baker et al., 2015). Then, NLSs of heterogeneous nuclear ribonucleoprotein (hnRNP) D and transporter associated with antigen processing (TAP) proteins binding TNPO1 with K_D_ values of 3.2 and 17 nM, respectively (Imasaki et al., 2007). Finally, the N-terminal tail of histone H3 was shown to bind seven importins with a preference for TNPO1 and IPO5 with K_D_ values of 77 and 57 nM, respectively (Soniat and Chook, 2016; Soniat et al., 2016).

We tested selected NLSs for their nuclear transport dynamics using the developed OT in live-cell imaging, as described above. For faster turn-around, we first performed screening in neuroblastoma N2a cells, which showed MycA9 import kinetics close to that observed in neurons (Supplementary Figure S4). All variants showed nuclear localization of mCherry in darkness and its export to the cytoplasm upon NIR light illumination (Figure 3C). This relocalization was slightly less efficient for MeCP2 and NiV W NLSs. While nuclear recovery of the QPAS1-cargo fusions in darkness was inefficient within 40 min for MycA9 NLS (Figure 3C), MeCP2 and NiV W showed almost complete re-localization by this time point. hnRNP D and TAP NLS variants showed partial nuclear recovery. The H3 NLS demonstrated almost no recovery, thus, we excluded this variant from further tests.

Next, we tested these constructs in cultured primary cortical neurons (Figure 3D). Again, MeCP2 and NiV W NLS variants showed less efficient relocalization to the cytoplasm after illumination, but more complete recovery at 30 min in darkness. The hnRNP D NLS showed complete relocalization to the cytoplasm but demonstrated slower recovery in darkness. Relocalization and recovery for TAP NLS were slower than observed in N2a and we excluded this NLS from further tests.

### Characterization of the Selected Uncommon NLSs in Neurons

**FIGURE 4:**
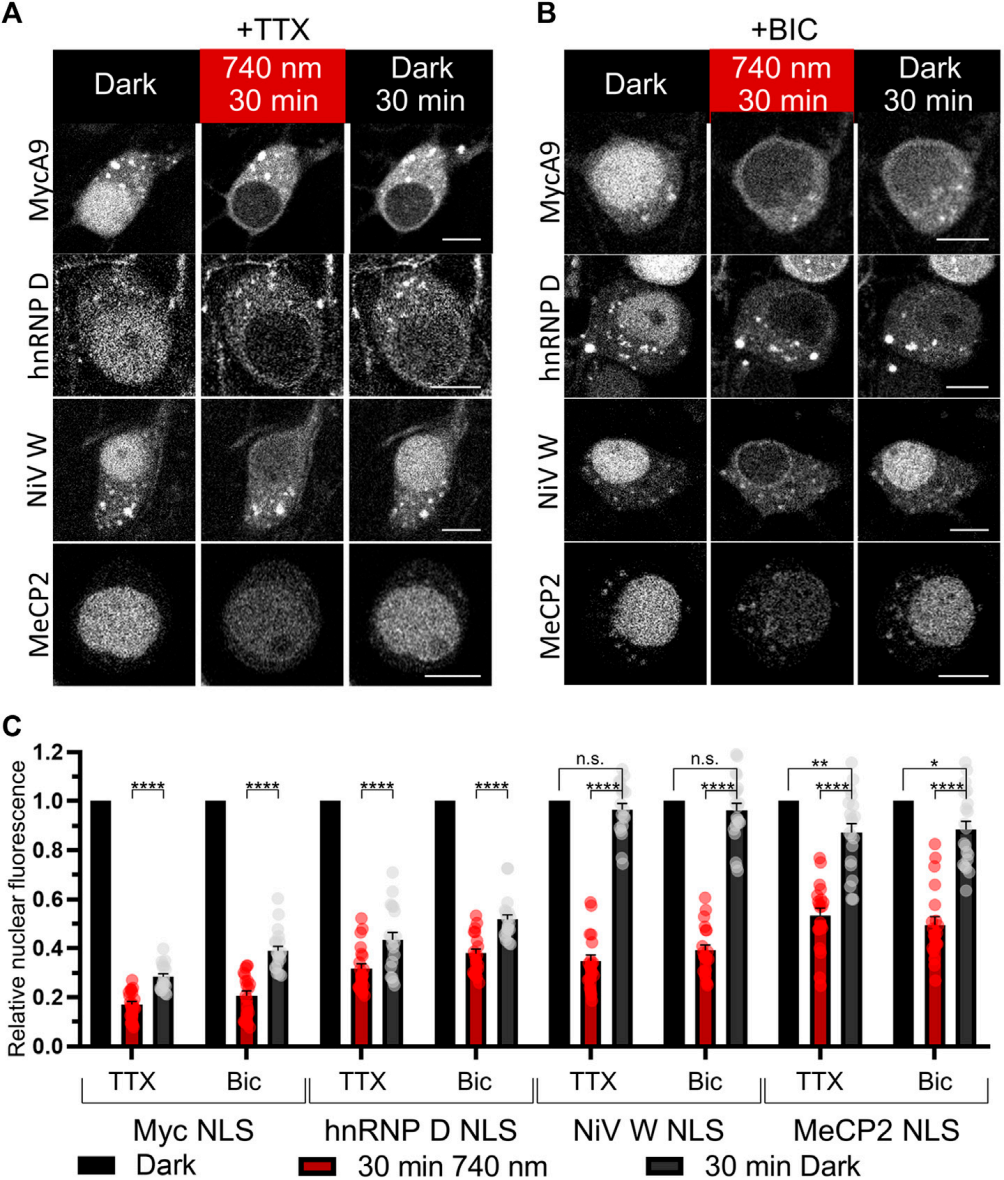
Characterization of selected NLSs with the NIR OT for light-controlled nuclear export in neurons. **(A,B)** The mCherry-labeled NC in living primary cortical neurons expressing CC and different NC variants were imaged with confocal microscopy at DIV 16. Cells were treated with 1 µM tetrodotoxin (TTX) to silence **(A)** or 50 µM bicuculline (BIC) to stimulate neural activity **(B)** and illuminated with NIR light (740 nm) for 30 min followed by 30 min of darkness. Representative images are shown. Scale bar, 10 μm. **(C)** Quantification of relative nuclear fluorescence before illumination, after 30 min of NIR light, and after subsequent 30 min in darkness. Mean values for individual cells ± S.E.M. were calculated (*n* = 20). Statistical significance was determined using one-way ANOVA and Tukey’s test. ****p < 0.0001, **p = 0.0043, *p = 0.0118, n. s.—no significance.

Since it is known that importin localization may be influenced by neuronal activity (Thompson et al., 2004; Jeffrey et al., 2009) we tested light-mediated export and import of the NC containing MycA9, MeCP2, NiV W, hnRNP D NLSs in silenced (1 µM tetrodotoxin (TTX)) (Figures 4A,C) or stimulated (50 µM bicuculline (BIC)) neurons (Figures 4B,C). Only MycA9 NLS showed a low but statistically significant difference demonstrating smaller nuclear fluorescence recovery under TTX treatment (Supplementary Figure S5A). This quantitative experiment performed using confocal microscopy also reported relocalization efficiencies of the OTs with selected NLSs (Figure 4C). 30 min illumination resulted in the cytoplasmic release of approximately 80% NC for MycA9, 60–70% for NiV W and hnRNP D, and 50% for MeCP2 NLSs. Then 30 min in darkness resulted in the recovery of nuclear fluorescence 30–40% of initial values for MycA9, 40–50% for hnRNP D, 90–100% for NiV W, and 90% for MeCP2 NLSs.

Then we tested NiV W and hnRNP D NLS variants, which demonstrated both efficient light-induced export and relatively fast nuclear recovery, in comparative time-lapse wide-field microscopy imaging as above (Supplementary Figure S5B,C). Light-induced nuclear export dynamics were similar between variants (*τ*_1/2_ = 3.85, 5.81, 4.65 min for NiV W, hnRNP D, and MycA9 NLSs, respectively). Subsequent nuclear import in darkness demonstrated more than twice faster nuclear import for NiV W NLS (*τ*_1/2_ = 10.38 min), compared to MycA9 (*τ*_1/2_ = 24.99 min). hnRNP D NLS also showed faster dynamics (*τ*_1/2_ = 18.50 min) than MycA9.

Thus, by varying NLSs, it is possible to adjust the dynamics and the extent of NIR light-controlled nuclear transport. NiV W NLS provided fast restoration of the initial system state in darkness, while originally tested MycA9 NLS allowed more efficient protein release from the nucleus accompanied by its slower return in darkness.

Additionally, we investigated how decreasing the NC size influences protein distribution. We substituted the linker between the cargo and mCherry with 2A self-cleaving peptide in the NCs with MycA9 and NiV W NLSs and tested their localizations in darkness and under NIR light in N2a cells using immunostaining (Supplementary Figure S5D,E). In darkness, the shorter NC with MycA9 (40.9 kDa) was distributed in various ratios between the nucleus and the cytoplasm (Supplementary Figure S5D). Under NIR light, it was fully exported to the cytoplasm. In contrast, the smaller NC with NiV W NLS (42.9 kDa) had similar distinct nuclear localization in darkness as the bigger NC (68.9 kDa) and was exported under NIR light (Supplementary Figure S5E). Therefore, NLSs influence both transport dynamics and the nucleocytoplasmic ratio, as expected.

### Optimization of NIR OT for Light-Induced Gene Expression in Neurons

**FIGURE 5:**
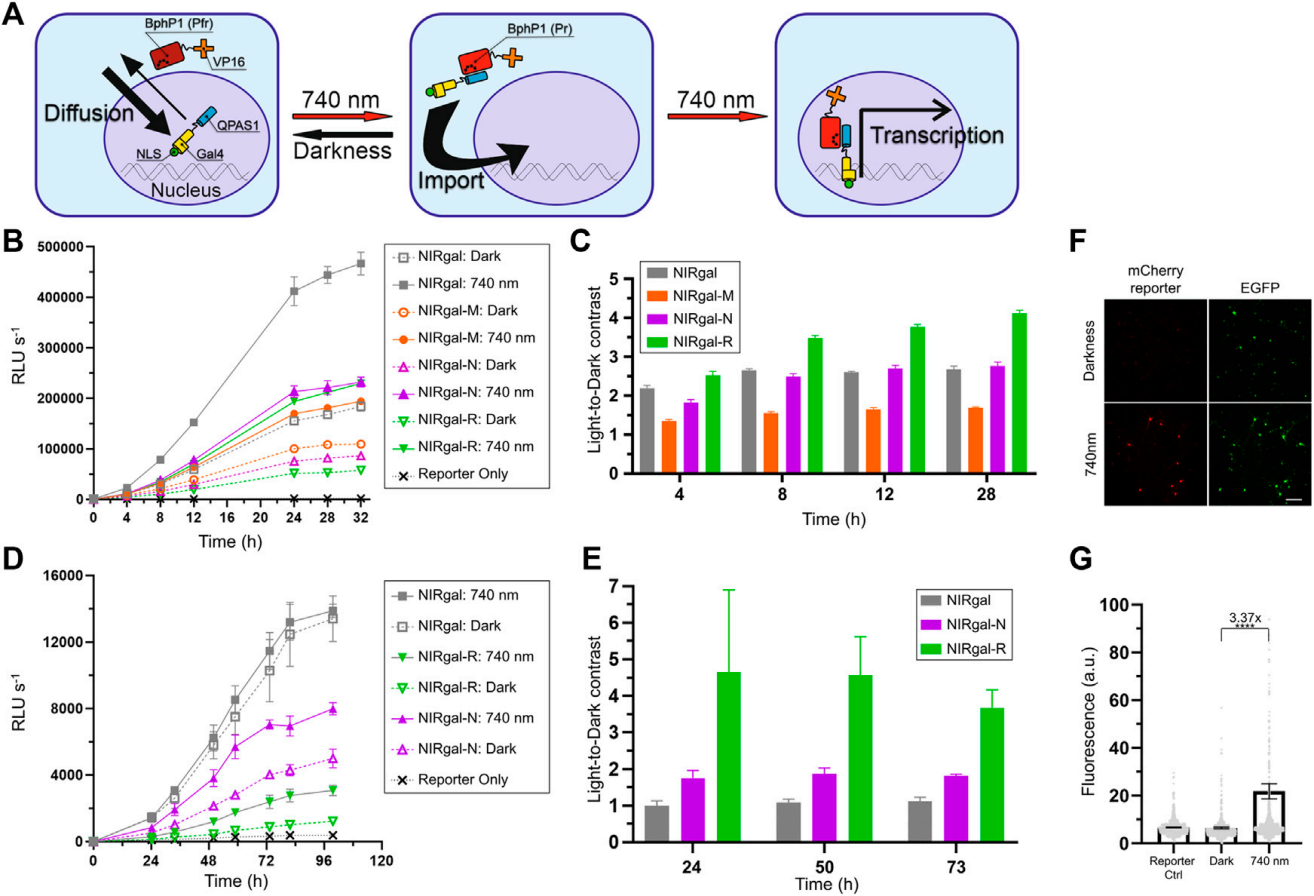
Optimization of NIR OT for light-induced gene expression in neurons. **(A)** Schematics showing how nuclear transport is used in the system. Bulky BphP1-VP16 fusion (97 kDa) is located in the cytoplasm while diffusing smaller QPAS1-Gal4-NLS (39 kDa) is actively imported to the nucleus. Upon illumination, the BphP1 component is pulled to the nucleus through binding to the QPAS1 resulting in the expression of a UAS-controlled reporter gene. **(B–E)** *Gaussia* luciferase reporter expression over time for the system variants containing the selected NLSs in N2a cells **(B,C)** and primary neurons **(D,E)** in response to 740 nm light. Raw signals **(B,D)** corresponding to different illumination time and calculated light-to-dark contrasts **(C,E)** are shown (*n* = 3; error bars are S.E.M.). RLU, relative light unit. **(F)** Performance of the optimized NIRgal-R system with UAS-mCherry reporter in cultured primary neurons. Cultured cortical neurons were transduced with AAV2 mixture containing NIRgal-R system, UAS-mCherry reporter, and CAG-EGFP as a transduction marker at DIV 6. At DIV 9, neurons were illuminated for 72 h with NIR light (740 nm) or kept in darkness. Fixed cells were imaged with widefield fluorescence microscopy. Scale bar, 100 μm. **(G)** Quantification of the experiment described in **(F)**. mCherry reporter fluorescence is calculated in individual fixed cells identified using combined DAPI and EGFP masks. Mean values for individual cells ± S.E.M are shown (*n* > 500.). ****p < 0.0001 by Tukey’s test.

We reasoned that the performance of NLS-containing OTs in neurons can be improved by selecting NLS with optimal kinetics. We decided to apply selected NLSs to the BphP1-QPAS1-based NIR light-induced Gal4/UAS gene expression system (Redchuk et al., 2018b). This OT consists of two components encoded in a single AAV: BphP1 fused to a VP16 transcriptional activation domain and QPAS1 fused to Gal4 DNA-binding domain and SV40G7 NLS (Figure 5A). We named this system as NIRgal. In darkness, the components are mostly separated by the nuclear membrane, since large BphP1-VP16 (97 kDa) does not diffuse to the nucleus and QPAS1-Gal4-NLS (39 kDa), which diffuses to the cytoplasm, is actively imported. Upon illumination, the components interact and are pulled to the nucleus to drive the expression of a UAS-controlled reporter gene. In contrast to the OT for the nuclear export developed above, the BphP1 component does not contain NES and can be imported to the nucleus in complex with the QPAS1-component. Therefore, NLS fused to QPAS1-GAL4 plays two roles in this OT. First, upon light illumination it is essential to pulling the assembled protein complex into the nucleus where the reporter DNA is located. Second, it is used to spatially separate reactive system components to different cellular compartments, thus limiting their background interaction in darkness. Such approach have been used in similar BphP1-based systems (Kaberniuk et al., 2016; Redchuk et al., 2018a) and photoactivatable split Cre recombinase (Meador et al., 2019). Therefore, the optimal NLS in NIRgal system should provide balance between active nuclear transport needed for strong system response and cytoplasmic availability to bind BphP1-VP16 component. To select such NLS, we performed experimental screening of NLSs with different properties.

We substituted SV40G7 NLS with MeCP2, NiV W, or hnRNP D NLSs in the NIRgal system producing NIRgal-M, NIRgal-N, and NIRgal-R, respectively. First, we tested these variants in N2a cells. We used secreted *Gaussia luciferase* (Gluc) as a reporter and monitored its expression over time using an assay with coelenterazine substrate. NIRgal-R showed the lowest dark background with a relatively high response to light (Figure 5B) providing the highest light-to-dark contrast (4.1-fold at 28 h) (Figure 5C **)**. NIRgal produced the highest response to illumination accompanied by a high background in darkness too. Then, we tested the systems with the selected NLSs in neurons using the Gluc reporter (Figures 5D,E). Again, NIRgal-R demonstrated the highest light-to-dark contrast (4.6-fold at 50 h), compared to the NIRgal and NIRgal-N systems, which showed less than 2-fold maximal contrasts over time. Additionally, we proved the functionality of the NIRgal-R system in cultured primary neurons with mCherry reporter (Figures 5F,G).

## Discussion

In this work, we provide direct experimental evidence that commonly used NLSs, such as Myc and SV40 preferentially binding KPNA2 importin, promote slower nuclear import in neurons than in non-neuronal cells. We identified uncommon NLSs preferentially binding KPNA3, KPNA4, and TNPO1 transporters, which mediate faster nuclear import, and applied them to the engineering of OTs for use in neurons.

First, we developed the NIR light-controlled OT for the nuclear transport of a protein of interest. Upon 740–780 nm illumination, the protein is exported to the cytoplasm within minutes. In the darkness, it is transported back to the nucleus in time depending on the utilized NLS (no more than 30 min). We used this OT for direct characterization of nuclear import kinetics mediated by NLSs with different transporter specificities.

For widely used Myc NLS, we observed a considerable difference in the kinetics of nuclear import in neuronal and non-neuronal cells (Figure 2). It correlated with the difference in expression levels of a respective nuclear transporter. Myc and related SV40 NLSs preferably bind KPNA2, which is depleted in neurons (Hosokawa et al., 2008; Hodge et al., 2019) and abundant in HeLa cells (Bekker-Jensen et al., 2017). Earlier studies reported a correlation between the importin expression and the nucleocytoplasmic ratio in yeast (Timney et al., 2006) and mammalian cell lines (Zannini et al., 2003; Riddick and Macara, 2005, 2007; Hazawa et al., 2020). We directly demonstrated that the nuclear import dynamics is also affected. Overexpression of KPNA2 should result in faster kinetics of nuclear import for KPNA2-dependent Myc and SV40 NLSs (Riddick and Macara, 2005, 2007; Timney et al., 2006). However, upregulation of KPNA2 in neurons was correlated with neuronal apoptosis (Xu et al., 2016) and, therefore, can significantly alter cellular physiology.

We used the developed OT to screen uncommon NLSs preferentially binding more abundant transporters KPNA3, KPNA4, and TNPO1 with high affinity, to identify NLSs that mediate fast and efficient nuclear transport in neurons. We found the NLSs that mediate considerably faster nuclear import than MycA9: NiV W and MeCP2 NLSs binding KPNA3 and KPNA4, and hnRNP D NLS binding TNPO1. Abundant KPNA3 and KPNA4 isoforms play important roles in neuronal cell physiology and their dysregulation was linked to neurodegenerative diseases (Moore et al., 2020; Pasha et al., 2021). KPNA4 regulates chronic pain pathways in peripheral sensory neurons by mediating c-Fos nuclear import (Marvaldi et al., 2020). KPNA3 and KPNA4 were also studied regarding the transport of TAR DNA-binding protein 43 (TDP-43) and the formation of aggregates of this protein in the central nervous system (Pasha et al., 2021), which is a hallmark of many neurodegenerative diseases (Jo et al., 2020).

While this work was performed on primary cortical neurons, we hypothesize that our results and optimized NLSs are applicable to all neuronal subtypes. Indeed, KPNA2 was found downregulated throughout the brain and spinal cord but with a moderate expression level in the olfactory bulb and reticular system (Hosokawa et al., 2008). We explored the Allen Mouse Brain Atlas RNA *in situ* hybridization database (https://mouse.brain-map.org/search/index) (Lein et al., 2007) for the data on importin expression measured in different mouse brain structures (Supplementary Figure S6). We found that TNPO1 specific to hnRNP D NLS showed the highest expression level across all brain areas, while KPNA2 and KPNA7 specific to Myc and SV40 NLSs (Miyamoto et al., 1997; Nadler et al., 1997; Oostdyk et al., 2020) were downregulated in most brain areas. KPNA4 specific to NiV W and MeCP2 NLSs demonstrated expression levels higher than KPNA2 in all brain structures.

We found that neuronal activity slightly influenced the efficiency of nuclear transport for MycA9 NLS, in contrast to the other selected NLSs (Supplementary Figure S5A). This effect can be explained by the previous observation that nuclear localization of some nuclear receptors including KNPA2 decreases upon silencing of neural activity and increases upon stimulation (Thompson et al., 2004; Jeffrey et al., 2009).

Using uncommon NLSs characterized here in neurons, we adjusted and optimized the performance of NLS-containing OTs in neurons. By changing NLS in the NIR OT for nuclear export, it was possible to regulate the extent of the nuclear export of a protein of interest as well as the dynamics of nuclear import in the darkness that shuts the system down.

By varying NLS in the NIR OT for light-induced gene expression, we developed the system that produced a reliable response to light and low dark background (up to >4-fold light-to-dark contrast) in neurons. hnRNP D NLS attached to the QPAS1-containing NC enabled an optimal balance between spatial separation of QPAS1 and BphP1-containing fusions and their accessibility for interaction.

The optimized NIR OT for gene expression can be activated by the most red-shifted light of 780 nm sensed by natural photoreceptors. It should enable its combinations with OTs and biosensors controlled by different wavelengths of light, such as LOV-based OTs, without crosstalk (Redchuk et al., 2017). Also, it can replace blue light-controlled OTs (Yamada et al., 2018, 2020) to avoid blue light-mediated toxicity (Marek et al., 2019) and off-target activation of immediate-early genes in neurons (Tyssowski and Gray, 2019).

This study suggests that various NLS-containing genetically encoded tools can be optimized for their use in neurons by replacing common NLSs with the NLSs selected in this work. Examples of OT systems that should benefit from NLS optimization include blue light OTs that use NLSs caged on the *As*LOV2 domain (Niopek et al., 2014; Yumerefendi et al., 2015). We observed previously that the blue light-activated *As*LOV2-NLS module of the iRIS tool (Redchuk et al., 2017) showed less efficient nuclear import in N2a cells and primary neurons, compared to non-neuronal cell lines (Redchuk et al., 2018b). iRIS uses LOV2-caged Myc NLS (with P1M substitution) binding KPNA2 depleted in neurons. Also blue light-controlled systems for gene expression that use CRISPR-dCas9 (Nihongaki et al., 2015, 2017; Polstein and Gersbach, 2015; Bubeck et al., 2018) or transcription activator-like effector (TALE) (Konermann et al., 2013) could be optimized in neurons by varying NLSs.

This study should draw more attention to uncommon NLSs and their functionality in neurons. So far, TNPO1-specific M9 NLS of hnRNP A1 was proven to be superior to SV40 for gene delivery in neurons (Ma et al., 2002). Utilizing neuron-specific NLSs instead of common ones should be useful in technologies for neuronal delivery of therapeutic proteins in virus-like particles (Banskota et al., 2022). Beyond OTs, it was shown that the efficiency of all CRISPR-based systems can be improved by increasing their nuclear translocation (Torres-Ruiz et al., 2017; Maggio et al., 2020). We predict that employing NLSs described here will be advantageous for applications of CRISPR-Cas systems in neuroscience (Heidenreich and Zhang, 2016) including recently developed neuron-optimized CRISPR/Cas9 genome editing systems (Fang et al., 2021), CRISPR-based transcriptional activation (Savell et al., 2019) and inhibition (Zheng et al., 2018) systems as well as CRISPR-Cas13 for silencing of neurodegeneration-associated genes in neurons (Powell et al., 2022). To summarize, uncommon neuron-specific NLSs, including NiV W, MeCP2, and hnRNP D characterized here, should become useful building blocks for synthetic biology applications in neurons.

## Materials and Methods

### Constructs and Cloning

All plasmids used in this study are listed in Supplementary Table S1. Annotated cDNA and amino acid sequences for optimized constructs are given in Supplementary Note S1.

For pAAV vectors designed in this work, we used short woodchuck hepatitis posttranscriptional regulatory element (WPRE3) and bovine growth hormone polyadenylation signal (bGHpA) (Choi et al., 2014). BphP1 and QPAS1 were cloned from Addgene plasmid #102584. All NLS and NES signals (see Supplementary Table S2) were cloned using synthesized oligonucleotides. Model cargo containing calmodulin-binding M13 peptide and C-terminal fragment of split tobacco etch virus protease was cloned from Addgene plasmid #92391.

The pAAV-U5-Gluc plasmid was generated by cloning the expression cassette to the pAAV backbone from the reporter vector pU5-Gluc (Wang et al., 2012). Then pAAV-U5-mCherry was obtained by substituting Gluc with mCherry.

DNA fragments for cloning were obtained using PCR amplification with Q5 DNA polymerase (New England Biolabs). Fragments were purified and concentrated using NucleoSpin Gel and PCR Clean-up Kit (Macherey-Nagel). Vector and fragments double-digestion and ligation were done using FastDigest restriction enzymes (Thermo Scientific) and T4 DNA Ligase (Thermo Scientific). Ligated plasmid products were introduced to TOP10 *Escherichia coli* cells using heat-shock transformation. Plasmids were purified using the NucleoSpin Plasmid Mini kit (Macherey-Nagel). Relevant genetic components were confirmed by Sanger sequencing (FIMM Genomics, University of Helsinki, Finland).

### Cell Culture and Transfection

Human embryonic kidney 293T cells (CRL-1573, ATCC), human epithelioid cervix carcinoma cells (HeLa; CCL-2, ATCC), mouse neuroblastoma Neuro-2a cells (N2a; CCL-131, ATCC) were cultured in Dulbecco’s modified Eagle’s medium (DMEM; 41,965-039, Gibco) supplemented with 10% (vol/vol) fetal bovine serum (FBS; 10270106, Gibco) and 1% (vol/vol) Antibiotic-Antimycotic (15240062, Gibco). All cell lines were cultured at 37 C and 5% CO_2_.

293T and N2a cells were transfected with Lipofectamine 2000 transfection reagent (11668027, Invitrogen). HeLa cells were transfected using Effectene (301427, Qiagen) or PEI transfection (24765, Polysciences).

For cell lines transfected with BphP1-based constructs, BV (FSIB655-9, CymitQuimica) was supplemented to the culture medium at 25 µM concentration. For primary neurons, we used 5–25 µM BV.

### Primary Neuron Culture

Primary rat cortical neuronal cultures were prepared at the Neuronal Cell Culture Unit (University of Helsinki) accordingly to the protocol (Sahu et al., 2019). All animal work was performed following the ethical guidelines of the European Convention and regulations of the Ethics Committee for Animal Research of the University of Helsinki.

Dissociated cortical neurons were plated in 1,050 cells/mm^2^ density to 35 mm Cellview glass-bottom dishes (627860, Greiner) for live-cell imaging, 12-well plate plastic coated with poly-l-lysine (0.5 mg/ml solution in 0.1 M borate buffer (Beaudoin et al., 2012), P2636, Sigma–Aldrich) for testing of NIR light-regulated gene expression system with Gluc reporter, and on 12 mm glass coverslips (in 24-well plate) coated with poly-l-lysine (as above) for testing of NIR light-regulated gene expression system with mCherry reporter. Cells were cultured in a complete neuronal media (CNM): neurobasal medium (12348017, Gibco) supplemented with 2% (vol/vol) B-27 Plus Supplement (A3582801, Gibco), 1% (vol/vol) GlutaMAX Supplement (35050061, Gibco), and 1% (vol/vol) penicillin-streptomycin (15140122, Gibco). One-third of culture media was renewed every 3 days, cells were maintained at 37 C and 5% CO_2_.

### AAV Preparation

AAV particles for delivery of genetic constructs to primary cultured neurons were prepared according to the protocol described in (Challis et al., 2019). All particles have an AAV2 serotype. The pDG plasmid combining pHelper plasmid and AAV2 capsid encoding plasmid was obtained as a gift from the AAV Core Facility of the University of Helsinki. AAV2 bearing CAG-EGFP was purchased from the AAV Core Facility of the University of Helsinki.

### Fluorescence Microscopy

Widefield microscopy was performed using an Olympus IX83 inverted epifluorescence microscope equipped with a Xenon arc lamp (Lambda LS, Sutter). An ORCA-Flash4.0 V3 (Hamamatsu) camera was used for image acquisition. Cells were imaged using either a 20 × 0.75 NA air or a 60 × 1.35 NA oil objective lens (UPlanSApo, Olympus). HEK293T, HeLa, and N2a cells were imaged using 35 mm Cellview glass-bottom dishes (627860, Greiner) with a Live Cell Imaging Solution (Invitrogen, A14291DJ) in a humidified 37 C atmospheric chamber (Okolab). All images were captured using SlideBook (Intelligent Imaging Innovations) software.

Confocal imaging was performed with a Leica TCS SP8 microscope equipped with a 63 × 1.2 NA water (HC PL APO CS2) objective lens and several lasers (405, 488, 561, 594, and 633 nm), PMT, and HyD detectors. Images were captured using LAS X (Leica) software.

Cultured primary neurons were imaged in HEPES-based Tyrode’s solution (5 mM KCl, app. 120 mM NaCl, 1 mM MgCl_2_, 1.8 mM CaCl_2_, 1.04 mM Na_2_HPO_4_, 26.2 mM NaHCO_3_, 10.9 mM HEPES, and 10 mM d-glucose) pH 7.4. The osmolarity of Tyrode’s solution was adjusted with NaCl to match present neuronal culture media using Micro-Osmometer Model 3,320 (Advanced Instruments).

### NIR Light-Induced Export and Nuclear Import Dynamics

For capturing NIR light-induced export, NIR (3 mW/cm^2^) illumination was applied using the custom-assembled 740/25 nm LED array (LED Engin). The light intensity was measured by a power meter (PM100D, Thorlabs).

We used the 1:2 NC:CC plasmid or AAV particles ratio for cell lines transfection and primary neurons transduction respectively, if not stated otherwise.

AAV transduction of cultured primary neurons was done at 6 days *in vitro* (DIV). We used a total multiplicity of infection (MOI) of 7.5 × 10^4^ vg/cell. The imaging was done at DIV 10–16.

Z-stacks at multiple fields of view (FOVs) were captured at regular time intervals. Afterward, using Fiji ImageJ (Schindelin et al., 2012) we compiled time-lapse stacks selecting appropriate Z-planes for each time point to counter possible cell or FOV drift and performing stack alignment with Linear Stack Alignment with SIFT plugin. Photobleaching was corrected using a simple ratio method. To measure the dynamics of fluorescent intensity, the region of interest (ROI) for each cell was set on the cell nucleus avoiding nuclear membrane and nuclei for each timepoint. The background fluorescence was subtracted from mean fluorescence values for each cell, and then fluorescent intensity was normalized to the initial value. Graphs plotting and relocalization half-time values calculation with the nonlinear fitting was done using Prism 8 (GraphPad).

### Testing NIR Light-Regulated Reporter Expression in Mammalian Cells

NIR light-regulated gene expression system variants have been tested in N2a cells and cultured primary cortical neurons.

N2a cells were transfected using Lipofectamine 2000 (11668027, Invitrogen), with the system-encoded plasmid and Gluc reporter plasmid. All samples were triplicated. 10 µM BV was added to DMEM media supplemented with 10% (vol./vol.) FBS. 12–24 h after transfection the media was renewed. Then half of the samples were left in darkness (in a non-transparent ventilated box) and another half were illuminated. Cells were illuminated directly in a CO_2_ incubator with a 740/25 nm LED array (LED Engin) at 1 mW/cm^2^ and a 25% duty cycle: 30 s of light and 90 s of darkness. Supernatant samples were collected at selected time points and stored at −20°C.

Cultured primary neurons were transduced at DIV6 with AAV2 mixture encoding the system variant and Gluc or fluorescent protein reporter in 2:1 system:reporter ratio (MOI 0.5 × 10^5^ vg/cell). 25 µM BV was added to the media. At DIV9 the third of the media was renewed, and the experiment proceeded as described above for N2a. Collected supernatant samples containing secreted Gluc reporter were diluted in 5 mM NaCl PBS in 96-well half-area white plates (Costar). Then 5 µM coelenterazine (NanoLight Technology) was added and bioluminescence signals were immediately measured using Victor X3 multilabel plate reader (PerkinElmer). The light-to-dark contrast was calculated as a ratio of luminescence signals of the illuminated sample to the sample kept in dark [values from the starting point were subtracted from respective samples; background signal generated by reporter alone (Reporter Only control sample) was subtracted from both signals values].

For the system characterization with microscopy, we used UAS-mCherry AAV as a reporter. Additional CAG-EGFP AAV was used as a transduction marker. Neurons were fixed after 3 days of NIR illumination (the same regimen as above) or darkness and imaged using Olympus IX83 microscope with 20 ×0.75 NA air objective lens. To calculate the response, more than ten FOVs of fixed neuron samples were captured in three channels: DAPI (excitation filter: 387/11 nm; emission filter: 480/40 nm), EGFP reporter (ex.f.: 485/20 nm; em.f.: 525/30 nm), and mCherry coexpressed protein (ex.f.: 560/25 nm; em.f.: 607/36 nm). For each sample, captured FOVs were processed in Fiji ImageJ (Schindelin et al., 2012) as follows. For DAPI and EGFP channels, separate masks were created using the AutoThreshold Triangle algorithm, which was then segmented using the Watershed algorithm. For DAPI, the segmented particles were limited by size (120–400 μm^2^) and circularity (0.60–1.00) to eliminate debris and artefacts. Then, DAPI and EGFP masks were combined with the “AND create” function in DAPI & EGFP mask selecting nuclei of neurons expressing the EGFP marker (<120 μm^2^ particles were excluded). The resulting mask was applied to the mCherry channel to measure the mean mCherry reporter fluorescence signal for each neuron. The resulting individual and mean fluorescence values were plotted using Prism 8 (GraphPad). The background signal in mCherry channel (quantified on the sample without mCherry reporter) was subtracted from values for all samples and the light-to-dark ratio was calculated.

### Fixed Samples Preparation and Immunostaining

Cultured primary neurons were fixed with 4% paraformaldehyde (Pierce, 28908) supplemented with 4% of sucrose for 30 min at RT, washed three times with PBS, and mounted using ProLong Glass Antifade Mountant with NucB (P36981, Invitrogen).

For immunostaining of N2a cells (Supplementary Figures S5D,E), cells were fixed with 4% paraformaldehyde supplemented with 4% of sucrose for 30 min at RT, washed three times with ice-cold PBS, permeabilized for 5 min with methanol at −20°C, washed twice with ice-cold PBS and blocked with 2% BSA, 22.52 mg/ml glycine PBS for 1 h RT. Then samples were incubated with mouse-anti-HA antibodies (sc-7392; Santa-Cruz) 1:150 in 2% BSA wash buffer (PBS with 0.1% Tween 20) for 1 h RT. After wash, samples were incubated with goat anti-mouse-AlexaFluor488 (A32723; Invitrogen) 1:1,000 in 2% BSA wash buffer for 1 h RT. Finally, samples were washed three times with the wash buffer and mounted using ProLong Glass Antifade Mountant with NucB (P36981, Invitrogen).

### Statistical Analysis

Data acquisition and processing are described above for each experiment. Statistical significance was determined using one-way ANOVA and Tukey’s test in Prism 8 (GraphPad), and significance was assigned at ^∗∗∗∗^ *p* < 0.0001, ^∗∗∗^ *p* < 0.001, ^∗∗^ *p* < 0.01, ^∗^ *p* < 0.05. The number of data points (*n*) and *p* values are indicated in the figures or figure legends.

## Data Availability

The original contributions presented in the study are included in the article/Supplementary Materials, further inquiries can be directed to the corresponding author.
