# Supplementary material for: Nuclear Localization Signals for Optimization of Genetically Encoded Tools in Neurons
Source: Front Cell Dev Biol. 2022 Jul 19;10:931237. doi: 10.3389/fcell.2022.931237 (PMC9344056; doi:10.3389/fcell.2022.931237)
Supplement: Supplementary file 1 [file Presentation1.pdf]

Nuclear Localization Signals for Optimization of Genetically Encoded Tools in Neurons

Supplementary Information

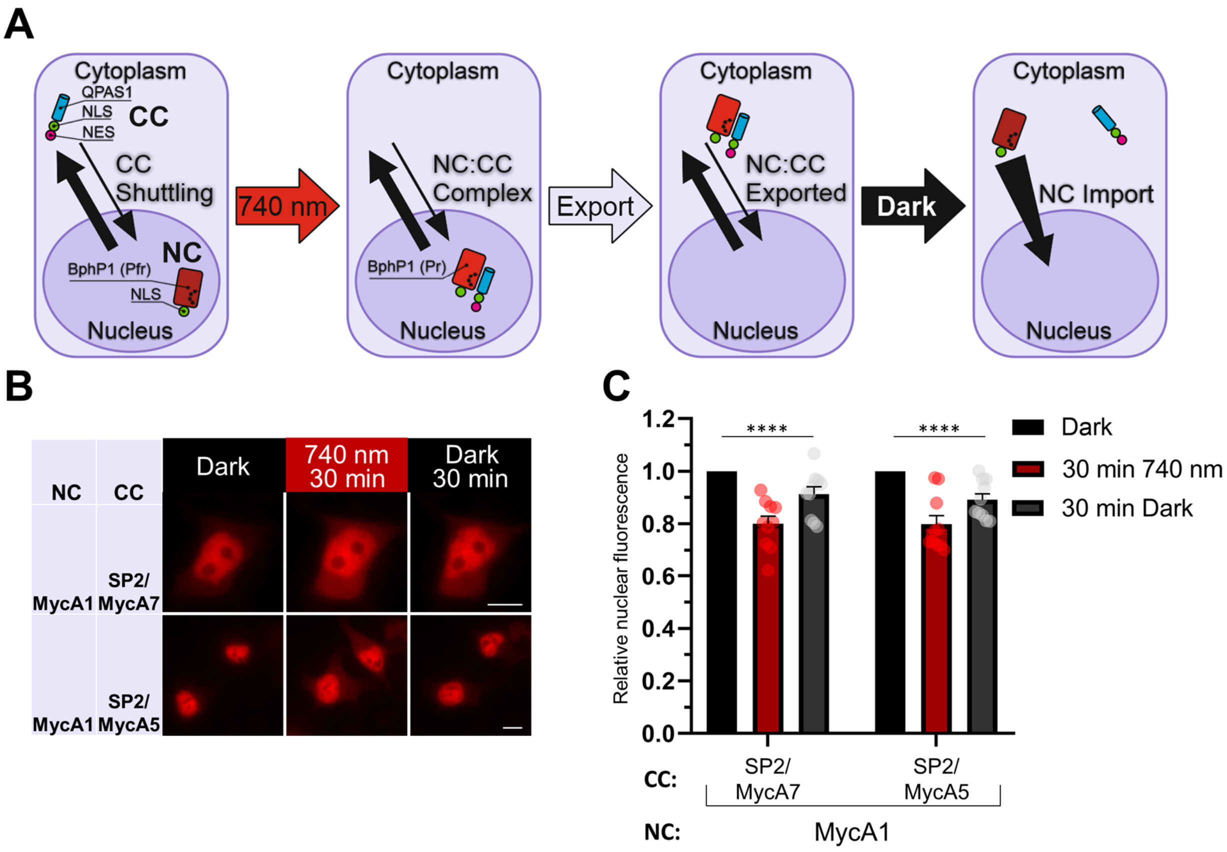

Supplementary Figure S1. NIR OT for light-controlled nuclear export with BphP1-based nuclear component. (**A**) Model of interaction for the NIR OT design with the BphP1-based nuclear component (NC) and QPAS1-based cytoplasmic component (CC). The NC is sequestered in the nucleus. The CC is mostly cytoplasmic due to a strong NES and it shuttles to the nucleus due to fused NLS. Upon NIR (740 nm) light illumination, the NC binds the CC in the nucleus, and the complex is pulled to the cytoplasm due to the strong NES on the CC. In darkness, the NC:CC complex dissociates, and the NC is imported back to the nucleus. (**B**) Representative images corresponding to the distribution of the NC component (internal BphP1 fluorescence with 685/20 nm excitation and 725/40 nm emission; 293T cells), captured with wide-field fluorescence microscopy in living cells. Points before illumination, after 30 min of 740 nm light illumination, and after subsequent 30 min in darkness are shown. Scale bar, 10 µm. (**C**) Quantification of relative nuclear fluorescence of the NC for the NC-CC combinations displayed on (**B**). Mean values for individual cells ±S.E.M. were calculated (n=10). Statistical significance was determined using one-way ANOVA test. ****$P < 0.0001$. SP2 – Super-PKI-2 NES; HIV – HIV-1 Rev NES.

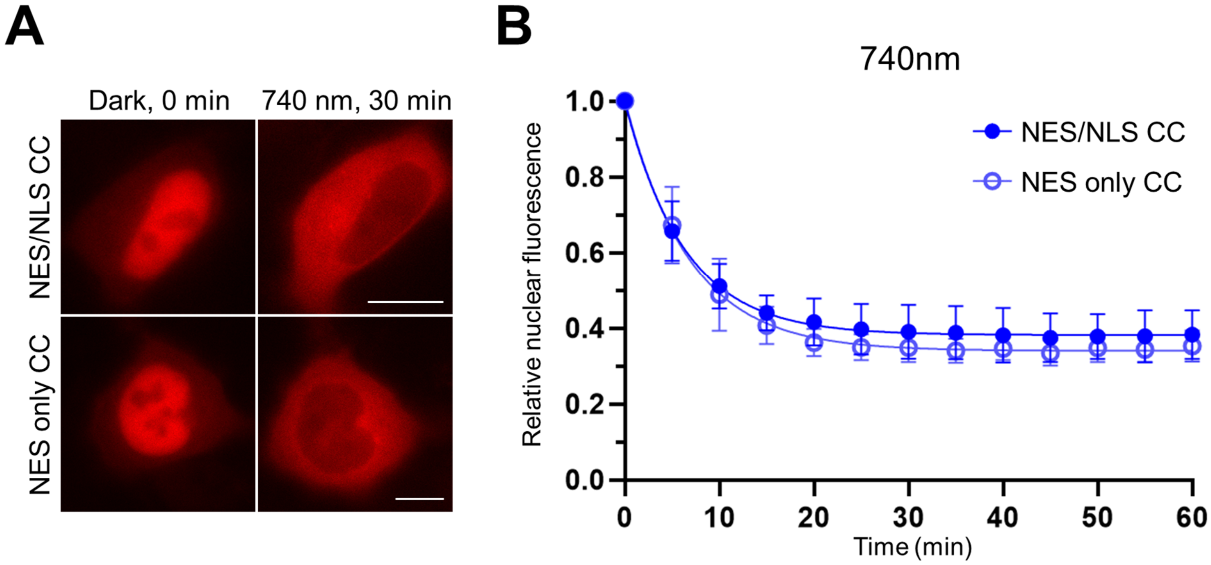

Supplementary Figure S2. The presence of MycA1 NLS in the BphP1-based cytoplasmic component (CC) does not influence the performance of the NIR light-controlled OT for nuclear export. (**A**) Representative images corresponding to the distribution of the QPAS1-based nuclear component (NC) fused to mCherry in HeLa cells with two types of CC. Scale bar, 10 µm. (**B**) Transport of the mCherry-containing NC was measured as a relative nuclear fluorescence upon NIR light illumination in HeLa cells. Mean values ±SD were calculated for individual cells (n=10) captured with wide-field fluorescence microscopy in living cells. Calculated half-times for NES/NLS CC and NES only CC respectively: 4.4 ± 0.6 and 4.7 ± 0.4 (± S.E.M.) min.

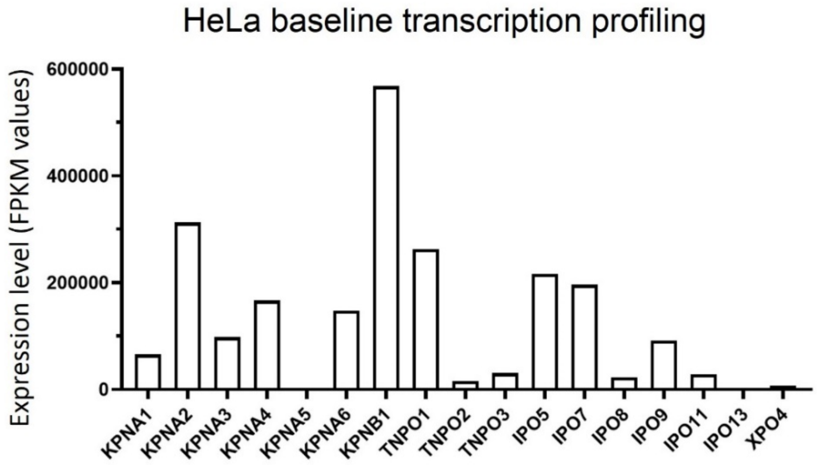

Supplementary Figure S3. Baseline transcriptome profiling for karyopherins in HeLa cells. Data are grouped by importin gene. Bars represent mean gene expression values. The source data (Bekker-Jensen et al., 2017) are available at the Expression Atlas database (https://www.ebi.ac.uk/gxa/home).

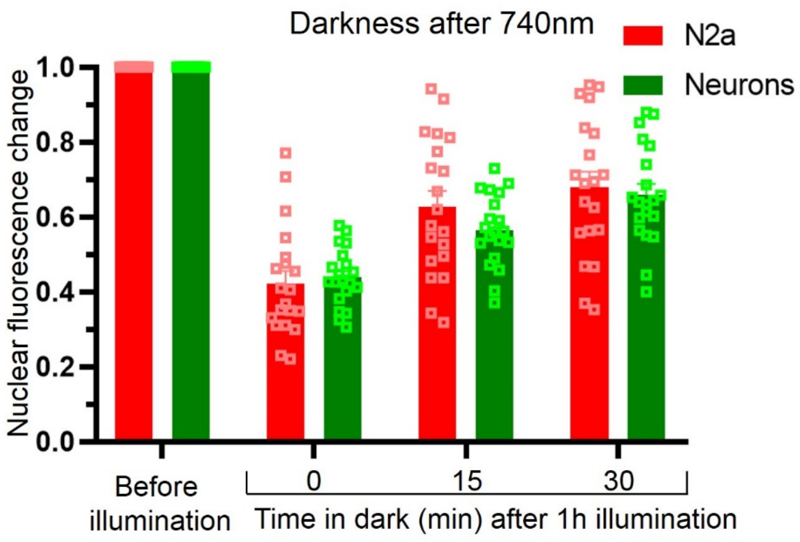

Supplementary Figure S4. Nuclear transport dynamics for MycA9 NLS observed in N2a cells, compared to neurons. Comparison of re-localization dynamics of the mCherry-containing nuclear component, measured as a relative nuclear fluorescence, in N2a cells and cultured primary neurons. Cells expressing NIR light-controlled OT for nuclear export were illuminated with NIR light (740 nm) for 1 h and then kept in darkness for 30 min while captured in live-cell imaging at indicated time points. Measured values for individual cells and calculated mean values ±S.E.M. (n=20 each) were normalized to the nuclear fluorescence measured before illumination.

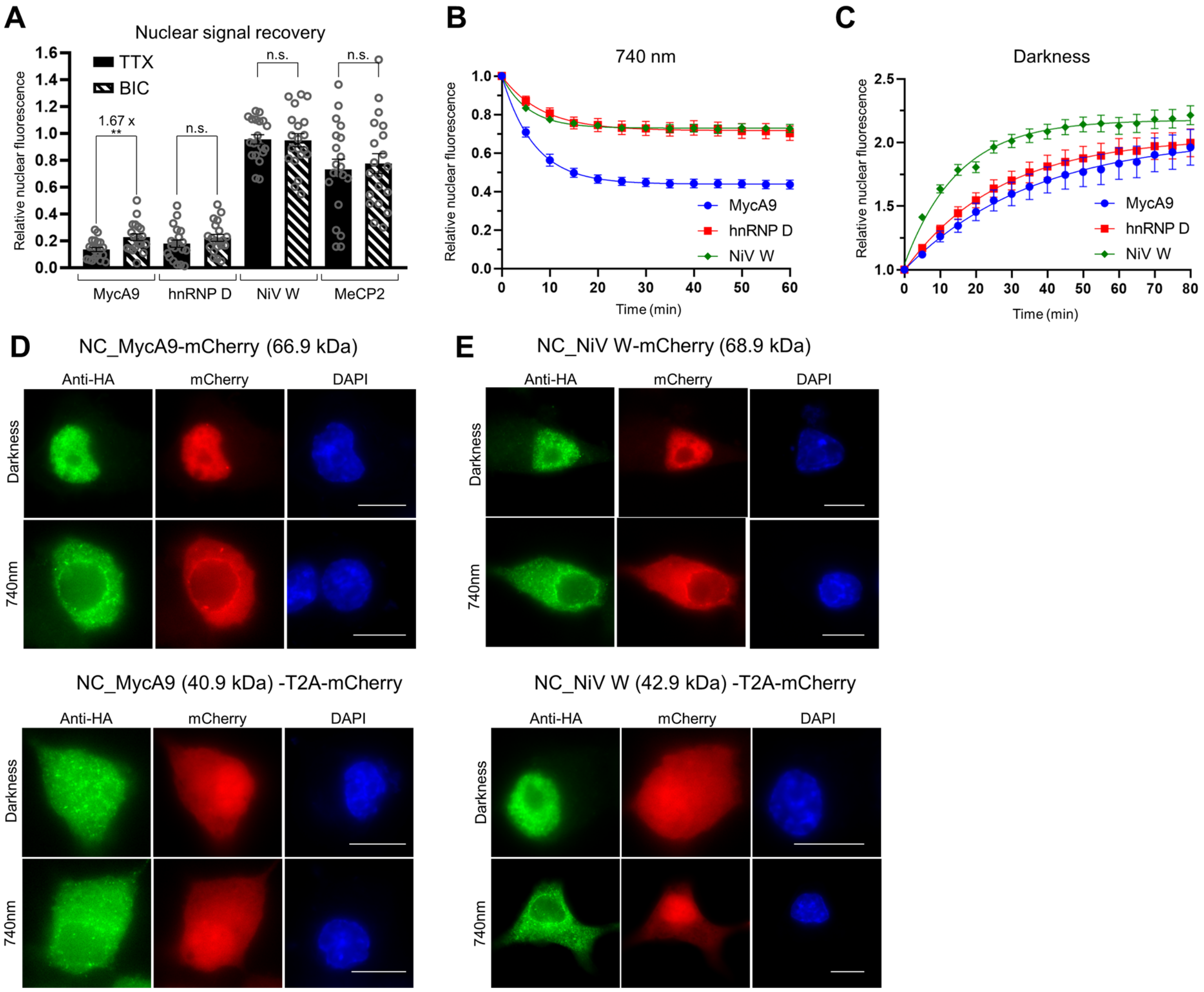

Supplementary Figure S5. Additional characterization of selected NLSs using the NIR OT for light-controlled nuclear export in primary neurons at DIV 12 and N2a cells. (**A**) Nuclear signal recovery quantification for Figure 4C is calculated as a ratio between nuclear fluorescence increase in darkness and preceding nuclear fluorescence decrease under NIR (740 nm) light $((F_{dark}-F_{NIR})\div(F_{NIR}-F_0))$ (n = 20; error bars are S.E.M.). $^{**}P = 0.0031$ by Welch's t-test; n.s. – no significance. (**B,C**) Relocalization dynamics for the mCherry-containing nuclear component (NC) measured as a relative nuclear fluorescence upon NIR light illumination (**B**) and subsequent darkness (**C**) in primary neurons transduced with NC and CC as 1:1. The NC contained either NiV W, or hnRNP D, or MycA9 NLSs. Calculated half-times (**B**) for NiV W, hnRNP D, and MycA9 NLSs respectively: 3.85 ± 0.57, 5.81 ± 1.44, and 4.65 ± 0.35 min. Calculated half-times (**C**) for NiV W, hnRNP D, and MycA9 NLSs respectively: 10.38 ± 0.95, 18.50 ± 3.55, and 24.99 ± 10.62 min. Mean values ± S.E.M. were calculated for individual cells (n=20 each) captured with wide-field fluorescence microscopy in live-cell imaging. (**D,E**) Representative images of the NC distribution and relocalization in N2a cells expressing NIR OTs for light-controlled nuclear export with either MycA9 (**D**) or NiV W (**E**) NLSs. The NC contains fused mCherry (upper panels) or does not contain it (lower panels) when mCherry is expressed separately through a self-cleaving 2A sequence. Representative images in darkness and after 40 min NIR illumination are shown. Fixed N2a cells were immunostained with anti-HA antibodies to detect NCs. Scale bar, 10 µm.

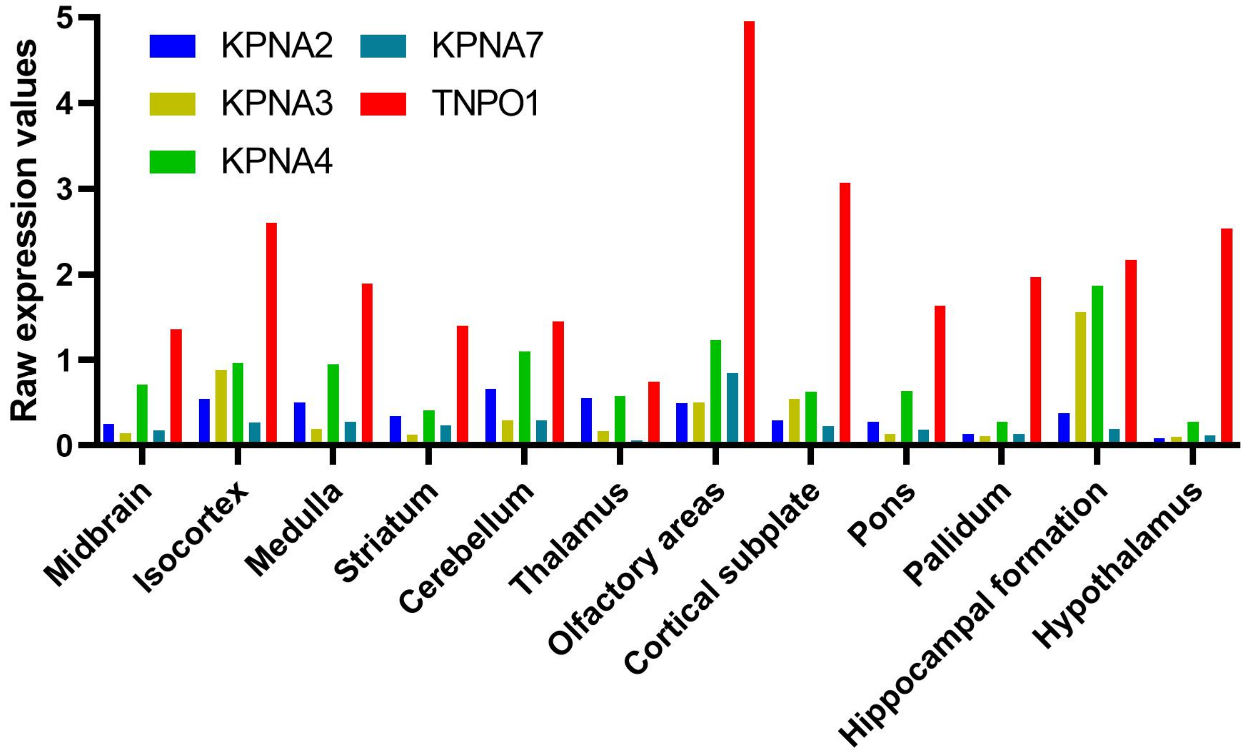

Supplementary Figure S6. Gene expression profiles for importins specific to NLSs studied in this work measured in different mouse brain structures. Data are grouped by importin gene. Bars represent mean gene expression values. Data on expression of respective genes were obtained from Allen Mouse Brain Atlas RNA *in situ* hybridization database (mouse.brain-map.org) (Lein et al., 2007)**.**

REFERENCES

Bekker-Jensen, D. B., Kelstrup, C. D., Batth, T. S., Larsen, S. C., Haldrup, C., Bramsen, J. B., et al. (2017). An Optimized Shotgun Strategy for the Rapid Generation of Comprehensive Human Proteomes. *cels* 4, 587-599.e4. doi: 10.1016/j.cels.2017.05.009.

Lein, E. S., Hawrylycz, M. J., Ao, N., Ayres, M., Bensinger, A., Bernard, A., et al. (2007). Genome-wide atlas of gene expression in the adult mouse brain. *Nature* 445, 168–176. doi: 10.1038/nature05453.

Redchuk, T. A., Karasev, M. M., Omelina, E. S., and Verkhusha, V. V. (2018). Near-Infrared Light-Controlled Gene Expression and Protein Targeting in Neurons and Non-neuronal Cells. *Chembiochem* 19, 1334–1340. doi: 10.1002/cbic.201700642.

Supplementary Table S1. Plasmids.

Plasmid Name	Plasmid backbone	Promoter	Features	Function	Reference	Used in Figures
pAAV-NQ9C	pAAV	hSyn	NLS(MycA9)-QPAS1-HA-M13-CTEV-mCherry	Nuclear component of the NIR light-induced export tool	This work	Fig. 2; 3; 4 Suppl.Fig. S4, S5
pCMV-NQ9C	pEGFP	CMV	NLS(MycA9)-QPAS1-HA-M13-CTEV-mCherry	Nuclear component of the NIR light-induced export tool	This work	Fig. 1, 2; Suppl.Fig. S2
pAAV-SSB1*	pAAV	hSyn	NES(Super-PKI-2)-BphP1-NLS(MycA1)	Cytoplasmic component of the NIR light-induced export tool and the NIR light/neural activity co-detection system	This work	Fig. 2; 3; 4 Suppl.Fig. S2, S4, S5
pCMV-SSB1	pEGFP	CMV	NES(Super-PKI-2)-BphP1-NLS(MycA1)	Cytoplasmic component of the NIR light-induced export tool	This work	Fig. 1, 2; Suppl.Fig. S2
pCMV-NB1	pEGFP	CMV	NLS(MycA1)-BphP1-HA-M13-CTEV-T2A-mCherry	Nuclear component of the NIR light-induced export tool	This work	Suppl.Fig. S1
pCMV-SSQ7	pEGFP	CMV	NES(Super-PKI-2)-QPAS1-NLS(MycA7)	Cytoplasmic component of the NIR light-induced export tool	This work	Suppl.Fig. S1
pCMV-SSQ5	pEGFP	CMV	NES(Super-PKI-2)-QPAS1-NLS(MycA5)	Cytoplasmic component of the NIR light-induced export tool	This work	Suppl.Fig. S1
pCMV-NQ1C	pEGFP	CMV	NLS(MycA1)-QPAS1-HA-M13-CTEV-mCherry	Nuclear component of the NIR light-induced export tool	This work	Fig. 1
pCMV-SHB1	pEGFP	CMV	NES(HIV)-BphP1-NLS(MycA1)	Cytoplasmic component of the NIR light-induced export tool	This work	Fig. 1
pCMV-SHB7	pEGFP	CMV	NES(HIV)-BphP1-NLS(MycA7)	Cytoplasmic component of the NIR light-induced export tool	This work	Fig. 1
pCMV-SSB7	pEGFP	CMV	NES(Super-PKI-2)-BphP1-NLS(MycA7)	Cytoplasmic component of the NIR light-induced export tool	This work	Fig. 1
pCMV-SSB	pEGFP	CMV	NES(Super-PKI-2)-BphP1	Cytoplasmic component of the NIR light-induced export tool	This work	Suppl.Fig. S2
pAAV-NQMC	pAAV	hSyn	NLS(MeCP2)-QPAS1-HA-M13-CTEV-mCherry	Nuclear component of the NIR light-induced export tool	This work	Fig. 3, 4; Suppl.Fig. S5

pAAV-NQNC*	pAAV	hSyn	NLS(NiV_W)-QPAS1-HA-M13-CTEV-mCherry	Nuclear component of the NIR light-induced export tool	This work	Fig. 3, 4; Suppl.Fig. S5
pAAV-NQRC	pAAV	hSyn	NLS(hnRNP_D)-QPAS1-HA-M13-CTEV-mCherry	Nuclear component of the NIR light-induced export tool	This work	Fig. 3, 4; Suppl.Fig. S5
pAAV-NQTC	pAAV	hSyn	NLS(TAP)-QPAS1-HA-M13-CTEV-mCherry	Nuclear component of the NIR light-induced export tool	This work	Fig.3
pAAV-NQHC	pAAV	hSyn	NLS(H3)-QPAS1-HA-M13-CTEV-mCherry	Nuclear component of the NIR light-induced export tool	This work	Fig.3
pAAV-NQ9-mCh	pAAV	hSyn	NLS(MycA9)-QPAS1-HA-M13-CTEV-T2A-mCherry	Nuclear component of the NIR light-induced export tool	This work	Suppl.Fig.S5
pAAV-NQN-mCh	pAAV	hSyn	NLS(NiV_W)-QPAS1-HA-M13-CTEV-T2A-mCherry	Nuclear component of the NIR light-induced export tool	This work	Suppl.Fig.S5
pAAV-NIRgal	pAAV	CaMKII	BphP1-VP16-T2A-NLS (SV40delV)-GAL4-QPAS1	NIR light-induced gene expression tool	(Redchuk et al., 2018)	Fig.5
pAAV-NIRgal-M	pAAV	CaMKII	BphP1-VP16-T2A-NLS (MeCP2)-GAL4-QPAS1	NIR light-induced gene expression tool	This work	Fig.5
pAAV-NIRgal-N	pAAV	CaMKII	BphP1-VP16-T2A-NLS (NiV_W)-GAL4-QPAS1	NIR light-induced gene expression tool	This work	Fig.5
pAAV-NIRgal-R*	pAAV	CaMKII	BphP1-VP16-T2A-NLS (hnRNP_D)-GAL4-QPAS1	NIR light-induced gene expression tool	This work	Fig.5
pAAV-U5-Gluc*	pAAV	U5	U5-Gluc	Reporter	This work	Fig.5
pAAV-U5-mCherry	pAAV	U5	U5-mCherry	Reporter	This work	Fig.5
pAAV-CAG-EGFP	pAAV	CAG	EGFP	Reporter	This work	Fig.5

** Annotated sequences are given in Supplementary Note 1*

Supplementary Table S2. Nuclear localization (NLS) and export (NES) signals.

**Name**	**Sequence**
Niv W NLS	CLGRRVVQPGMFEDHPPTKKARVSMR
MeCP2 NLS	KRPGRKRKAEADPQAIPKKRGR
H3 N-terminal region with NLS	MARTKQTARKSTGGKAPRKQLATKAARKS
hnRNP D NLS	SGYGKVSRRGGHQNSYKPY
TAP NLS	VAMSDAQDGPRVRYNPYTTRPNRR
Myc NLS	PAAKRVKLD
SV40 NLS	PKKKRKV
HIV NES	LQLPPLERLTL
Super-PKI-2 NES	NIDELALKFAGLDL

Supplementary Note S1. Annotated amino acid and DNA sequences for optimized constructs.

pAAV-SSB1

NES(Super-PKI-2)-BphP1-NLS(MycA1)

Protein sequence

MNIDELALKFAGLDLEFGGGGSGGGGSMVAGHASGSPAFGTADLSNCEREEIHLA
GSIQPHGALLVVSEPDHRIIQASANAAEFLNLGSVLGVPLAEIDGDLLIKILPHLDPTA
EGMPVAVRCRIGNPSTEYDGLMHRPPEGGLIIELERAGPPIDLSGTLAPALERIRTAG
SLRALCDDTALLFQQCTGYDRVMVYRFDEQGHGEVFSERHVPGLESYFGNRYPSS
DIPQMARRLYERQRVRVLVDVSYQPVPLEPRLSPLTGRDLDMSGCFLRSMSPIHLQ
YLKNMGVRATLVVSLVVGGKLWGLVACHHYLPRFIHFELRAICELLAEAIATRITALE
SFAQSQSELFVQRLEQRMIEAITREGDWRAAIFDTSQSILQPLHADGCALVYEDQIR
TIGDVPSTQDVREIAGWLDRQPRAAVTSTASLGLDVPELAHLTRMASGVVAAPISD
HRGEFLMWFRPERVHTVTWGGDPKKPFTMGDTPADLSPRRSFAKWHQVVEGTS
DPWTAADLAAARTIGQTVADIVLQFRAVRTLIAREQYEQFSSQVHASMQPVLITDA
EGRILLMNDSFRDMLPAGSPSAVHLDDLAGFFVESNDFLRNVAELIDHGRGWRGE
VLLRGAGNRPLPLAVRADPVTRTEDQSLGFVLIFSDATDRRTADAARTRFQEGILAS
ARPGVRLDSKSDLLHEKLLSALVENAQLAALEITYGVETGRIAELLEGVRQSMLRTAE
VLGHLVQHAARTAGSDSSSNGSQNKKASSAGGSAGGSAGGSAGGGSKLAAAKR
VKLDE*

pAAV-SSB1

ITR-hSyn-NES(Super-PKI-2)-BphP1-NLS(MycA1)-WPRE3- bGHpA-ITR

DNA sequence

CCTGCAGGCAGCTGCGCGCTCGCTCGCTCACTGAGGCCGCCCGGGCGTCGGGCGACCTTTGGTCGCCCGGCCTC
AGTGAGCGAGCGAGCGCGCAGAGAGGGAGTGGCCAACTCCATCACTAGGGGTTCCTGCGGCCGCACGCGTTGT
ACAGTGTCTAGACTGCAGAGGGCCCTGCGTATGAGTGCAAGTGGGTTTTAGGACCAGGATGAGGCGGGGTGGG
GGTGCCTACCTGACGACCGACCCCGACCCACTGGACAAGCACCCAACCCCCATTCCCCAAATTGCGCATCCCCTA
TCAGAGAGGGGGAGGGGAAACAGGATGCGGCGAGGCGCGTGCGCACTGCCAGCTTCAGCACCGCGGACAGTG
CCTTCGCCCCCGCCTGGCGGCGCGCGCCACCGCCGCCTCAGCACTGAAGGCGCGCTGACGTCACTCGCCGGTCC
CCCGCAAACTCCCCTTCCCGGCCACCTTGGTCGCGTCCGCGCCGCCGCCGGCCCAGCCGGACCGCACCACGCGA
GGCGCGAGATAGGGGGGCACGGGCGCGACCATCTGCGCTGCGGCGCCGGCGACTCAGCGCTGCCTCAGTCTGC
GGTGGGCAGCGGAGGAGTCGTGTCGTGCCTGAGAGCGCAGTACCGGACTCAGATCTACCATGAACATTGATGAG
TTGGCCCTCAAATTCGCAGGACTCGACCTGGAATTCGGAGGCGGAGGCTCTGGCGGCGGGGGCTCCATGGTGGC
AGGTCATGCCTCTGGCAGCCCCGCATTCGGGACCGCCGATCTTTCGAATTGCGAACGTGAAGAGATCCACCTCGC
CGGCTCGATCCAGCCGCATGGCGCGCTTCTGGTCGTCAGCGAGCCGGATCATCGCATCATCCAGGCCAGCGCCA
ACGCCGCGGAATTTCTGAATCTCGGAAGCGTGCTCGGCGTTCCGCTCGCCGAGATCGACGGCGATCTGTTGATCA
AGATCCTGCCGCATCTCGATCCCACCGCCGAAGGCATGCCGGTCGCGGTGCGCTGCCGGATCGGCAATCCCTCC
ACGGAGTACGACGGTCTGATGCATCGGCCTCCGGAAGGCGGGCTGATCATCGAGCTCGAACGTGCCGGCCCGC
CGATCGATCTGTCCGGCACGCTGGCGCCGGCGCTGGAGCGGATCCGCACGGCGGGCTCGCTGCGCGCGCTGTG
CGATGACACCGCGCTGCTGTTTCAGCAGTGCACCGGCTACGACCGGGTGATGGTGTATCGCTTCGACGAGCAGG
GCCACGGCGAAGTGTTCTCCGAGCGCCACGTGCCCGGGCTCGAATCCTATTTCGGCAACCGCTATCCGTCGTCGG
ACATTCCGCAGATGGCGCGGCGGCTGTACGAGCGGCAGCGCGTCCGCGTGCTGGTCGACGTCAGCTATCAGCCG
GTGCCGCTGGAGCCGCGGCTGTCGCCGCTGACCGGGCGCGATCTCGACATGTCGGGCTGCTTCCTGCGCTCGAT
GTCGCCGATCCATCTGCAGTACCTGAAGAACATGGGCGTGCGCGCCACCCTGGTGGTGTCGCTGGTGGTCGGCG
GCAAGCTGTGGGGCCTGGTTGCCTGTCACCATTATCTGCCGCGCTTCATCCATTTCGAGCTGCGGGCGATCTGCG
AACTGCTCGCCGAAGCGATCGCGACGCGGATCACCGCGCTTGAGAGCTTCGCGCAGAGCCAGTCGGAGCTGTTC
GTGCAGCGGCTCGAACAGCGCATGATCGAAGCGATCACCCGTGAAGGCGATTGGCGCGCAGCGATTTTCGACAC
CAGCCAATCGATCCTGCAGCCGCTGCACGCCGACGGTTGCGCGCTGGTGTACGAAGACCAGATCAGGACCATCG
GTGACGTACCTTCCACGCAGGATGTTCGCGAGATCGCCGGGTGGCTCGATCGCCAGCCACGTGCGGCGGTGACC
TCGACCGCGTCGCTCGGTCTCGACGTGCCGGAGCTCGCGCATCTGACGCGGATGGCGAGCGGCGTGGTCGCGG
CGCCGATTTCGGATCATCGCGGCGAGTTTCTGATGTGGTTCCGCCCCGAGCGCGTCCACACCGTTACCTGGGGCG
GCGATCCGAAGAAGCCGTTCACGATGGGCGATACACCGGCGGATCTGTCGCCGCGGCGCTCCTTCGCCAAATGG
CATCAGGTTGTCGAAGGCACGTCCGATCCGTGGACGGCCGCCGATCTCGCCGCGGCTCGCACCATCGGTCAGAC
CGTCGCCGACATCGTGCTGCAATTCCGCGCGGTGCGGACACTGATCGCCCGCGAACAGTACGAACAGTTTTCGTC
CCAGGTGCACGCTTCGATGCAGCCGGTGCTGATCACCGACGCCGAAGGCCGCATCCTGCTGATGAACGACTCGT
TCCGCGACATGTTGCCGGCGGGGTCGCCATCCGCCGTCCATCTCGACGATCTCGCCGGGTTCTTCGTCGAATCGA
ACGATTTCCTGCGCAACGTCGCCGAACTGATCGATCACGGCCGCGGGTGGCGCGGCGAAGTTCTGCTGCGCGGC
GCAGGTAATCGCCCGTTGCCGCTGGCAGTGCGCGCCGATCCGGTGACGCGCACGGAGGACCAGTCGCTCGGCTT
CGTGCTGATCTTCAGCGACGCTACCGATCGTCGCACCGCAGATGCCGCACGCACGCGTTTCCAGGAAGGCATTCT
TGCCAGCGCACGTCCCGGCGTGCGGCTCGACTCCAAGTCCGACCTCTTGCACGAGAAGCTGCTGTCCGCGCTGG
TCGAGAACGCGCAGCTTGCCGCATTGGAAATTACTTACGGCGTCGAGACCGGACGCATCGCCGAGCTGCTCGAA
GGCGTTCGCCAGTCGATGCTGCGCACCGCCGAAGTGCTCGGCCATCTGGTGCAGCACGCGGCGCGCACGGCCG

GCAGCGACAGCTCGAGCAATGGCTCGCAGAACAAGAAGGCTAGCAGTGCTGGCGGTAGTGCTGGTGGGAGTGC
TGGCGGTAGTGCTGGTGGCGGCTCCAAGCTTGCTGCTGCCAAGAGAGTTAAGCTGGACGAGTAAGGTACCTCAA
CCTCTGGATTACAAAATTTGTGAAAGATTGACTGGTATTCTTAACTATGTTGCTCCTTTTACGCTATGTGGATACGC
TGCTTTAATGCCTTTGTATCATGCTATTGCTTCCCGTATGGCTTTCATTTTCTCCTCCTTGTATAAATCCTGGTTAGTT
CTTGCCACGGCGGAACTCATCGCCGCCTGCCTTGCCCGCTGCTGGACAGGGGCTCGGCTGTTGGGCACTGACAA
TTCCGTGGTGTTTATTTGTGAAATTTGTGATGCTATTGCTTTATTTGTAACCATCTAGCTTTATTTGTGAAATTTGTGA
TGCTATTGCTTTATTTGTAACCATTATAAGCTGCAATAAACAAGTTAACAACAACAATTGCATTCATTTTATGTTTCA
GGTTCAGGGGGAGATGTGGGAGGTTTTTTAAAGCGGCCGCAGGAACCCCTAGTGATGGAGTTGGCCACTCCCTC
TCTGCGCGCTCGCTCGCTCACTGAGGCCGGGCGACCAAAGGTCGCCCGACGCCCGGGCTTTGCCCGGGCGGCCT
CAGTGAGCGAGCGAGCGCGCAGCTGCCTGCAGG

pAAV-NQNC

NLS(NiV W)-QPAS1-*HA*-M13-CTEV-mCherry

Protein sequence

MTCLGRRVVQPGMFEDHPPTKKARVSMRRMSAGGSAGGSAGGSAGGSSRGKN
MQAVTELHSRLIAAQQAMERDYWRLRELETRYRLVFDAAADAVMIVSAGDMRIV
EANRAAVNAISRVERGNDDLAGRDFLAEVAAADRDAVRDMLAQVRQRGTALSVL
VHLGRYDRAWMLRGSLMSSERRQVFLLHFTPVTTTPAIDDDDKGVVASAADGAE
GASDDAEDGGGGSGGGGSKL*YPYDVPDYA*GGSSSRRKWNKTGHAVRAIGRLSSL
EGGGGSGGGGSGGGGIEMSSMVSDTSCTFPSSDGIFWKHWIQTKDGQCGSPLVS
TRDGFIVGIHSASNFTNTNNYFTSVPKNFMELLTNQEAQQWVSGWRLNADSVLW
GGHKVFMVTGGSGGGGSTSGGGGSGGGGSVSKGEEDNMAIIKEFMRFKVHMEG
SVNGHEFEIEGEGEGRPYEGTQTAKLKVTKGGPLPFAWDILSPQFMYGSKAYVKHP
ADIPDYLKLSFPEGFKWERVMNFEDGGVVTVTQDSSLQDGEFIYKVKLRGTNFPSD
GPVMQKKTMGWEASSERMYPEDGALKGEIKQRLKLKDGGHYDAEVKTTYKAKKP
VQLPGAYNVNIKLDITSHNEDYTIVEQYERAEGRHSTGGMDELYK*

pAAV-NQNC

ITR-hSyn-NLS(NiV W)-QPAS1-*HA*-M13-CTEV-mCherry-WPRE3- bGHpA-ITR

DNA sequence

CCTGCAGGCAGCTGCGCGCTCGCTCGCTCACTGAGGCCGCCCGGGCGTCGGGCGACCTTTGGTCGCCCGGCCTC
AGTGAGCGAGCGAGCGCGCAGAGAGGGAGTGGCCAACTCCATCACTAGGGGTTCCTGCGGCCGCACGCGTTGT
ACAGTGTCTAGACTGCAGAGGGCCCTGCGTATGAGTGCAAGTGGGTTTTAGGACCAGGATGAGGCGGGGTGGG
GGTGCCTACCTGACGACCGACCCCGACCCACTGGACAAGCACCCAACCCCCATTCCCCAAATTGCGCATCCCCTA
TCAGAGAGGGGGAGGGGAAACAGGATGCGGCGAGGCGCGTGCGCACTGCCAGCTTCAGCACCGCGGACAGTG
CCTTCGCCCCCGCCTGGCGGCGCGCGCCACCGCCGCCTCAGCACTGAAGGCGCGCTGACGTCACTCGCCGGTCC
CCCGCAAACTCCCCTTCCCGGCCACCTTGGTCGCGTCCGCGCCGCCGCCGGCCCAGCCGGACCGCACCACGCGA
GGCGCGAGATAGGGGGGCACGGGCGCGACCATCTGCGCTGCGGCGCCGGCGACTCAGCGCTGCCTCAGTCTGC
GGTGGGCAGCGGAGGAGTCGTGTCGTGCCTGAGAGCGCAGTACCGGACTCAGATCTACCATGACATGCCTCGGA
AGAAGAGTGGTTCAACCTGGAATGTTCGAGGATCATCCTCCAACTAAAAAGGCAAGGGTTTCAATGAGAAGAAT
GAGTGCTGGTGGGTCCGCCGGCGGGAGTGCTGGCGGTAGTGCCGGCGGTAGTTCTAGAGGCAAGAACATGCAG
GCGGTCACCGAGCTGCATTCCCGGCTGATCGCTGCGCAGCAGGCGATGGAGCGCGACTATTGGCGGTTGCGTGA
ATTGGAGACTCGCTACCGCCTGGTGTTCGACGCTGCCGCCGATGCGGTGATGATCGTCTCCGCCGGCGACATGC
GCATCGTCGAAGCCAACCGGGCGGCGGTGAATGCGATCAGCCGCGTCGAGCGCGGCAATGACGACCTTGCGGG
GCGTGATTTCCTCGCCGAAGTGGCGGCTGCCGATCGCGATGCGGTGCGCGACATGCTGGCCCAGGTGCGTCAGC
GCGGCACCGCACTCAGCGTCCTCGTTCATCTCGGCCGTTACGACCGCGCCTGGATGCTGCGCGGTTCGCTGATGT
CGTCCGAGCGTCGTCAGGTTTTCCTGCTGCACTTCACCCCGGTGACCACGACTCCCGCGATCGACGACGACGACA
AAGGTGTCGTTGCTTCTGCTGCCGACGGTGCAGAGGGCGCCTCAGACGACGCAGAGGATGGAGGAGGCGGATC
TGGCGGAGGGGGCTCCAAGCTT*TACCCTTACGATGTGCCCGATTACGCC*GGGGGCAGCTCATCACGTCGTAAGT
GGAATAAGACAGGTCACGCAGTCAGAGCTATAGGTCGGCTGAGCTCACTCGAAGGTGGCGGTGGCTCTGGAGGT
GGTGGGTCCGGAGGAGGCGGCATCGAGATGTCCAGCATGGTGAGCGATACTAGCTGTACCTTCCCATCATCTGA
CGGAATCTTCTGGAAGCACTGGATTCAGACTAAGGACGGCCAGTGTGGCAGCCCACTGGTGAGCACACGAGACG
GATTCATCGTGGGGATTCACAGCGCCTCCAACTTTACAAACACCAATAACTATTTCACCTCAGTGCCAAAGAACTT
TATGGAGCTGCTGACCAACCAGGAGGCCCAGCAGTGGGTGAGCGGGTGGCGCCTGAACGCCGATTCCGTGCTGT
GGGGCGGGCACAAGGTGTTTATGGTGACCGGTGGCAGCGGAGGTGGGGGCTCCACTAGTGGCGGCGGAGGGA
GCGGGGGTGGAGGCTCCGTGAGCAAGGGCGAGGAGGATAACATGGCCATCATCAAGGAGTTCATGCGCTTCAA
GGTGCACATGGAGGGCTCCGTGAACGGCCACGAGTTCGAGATCGAGGGCGAGGGCGAGGGCCGCCCCTACGA
GGGCACCCAGACCGCCAAGCTGAAGGTGACCAAGGGTGGCCCCCTGCCCTTCGCCTGGGACATCCTGTCCCCTC
AGTTCATGTACGGCTCCAAGGCCTACGTGAAGCACCCCGCCGACATCCCCGACTACTTGAAGCTGTCCTTCCCCG
AGGGCTTCAAGTGGGAGCGCGTGATGAACTTCGAGGACGGCGGCGTGGTGACCGTGACCCAGGACTCCTCCCTG
CAGGACGGCGAGTTCATCTACAAGGTGAAGCTGCGCGGCACCAACTTCCCCTCCGACGGCCCCGTAATGCAGAA
GAAGACCATGGGCTGGGAGGCCTCCTCCGAGCGGATGTACCCCGAGGACGGCGCCCTGAAGGGCGAGATCAAG
CAGAGGCTGAAGCTGAAGGACGGCGGCCACTACGACGCTGAGGTCAAGACCACCTACAAGGCCAAGAAGCCCG
TGCAGCTGCCCGGCGCCTACAACGTCAACATCAAGTTGGACATCACCTCCCACAACGAGGACTACACCATCGTG
GAACAGTACGAACGCGCCGAGGGCCGCCACTCCACCGGCGGCATGGACGAGCTGTACAAGTAAGGTACCTCAA
CCTCTGGATTACAAAATTTGTGAAAGATTGACTGGTATTCTTAACTATGTTGCTCCTTTTACGCTATGTGGATACGC
TGCTTTAATGCCTTTGTATCATGCTATTGCTTCCCGTATGGCTTTCATTTTCTCCTCCTTGTATAAATCCTGGTTAGTT
CTTGCCACGGCGGAACTCATCGCCGCCTGCCTTGCCCGCTGCTGGACAGGGGCTCGGCTGTTGGGCACTGACAA
TTCCGTGGTGTTTATTTGTGAAATTTGTGATGCTATTGCTTTATTTGTAACCATCTAGCTTTATTTGTGAAATTTGTGA

TGCTATTGCTTTATTTGTAACCATTATAAGCTGCAATAAACAAGTTAACAACAACAATTGCATTCATTTTATGTTTCA
GGTTCAGGGGGAGATGTGGGAGGTTTTTTAAAGCGGCCGCAGGAACCCCTAGTGATGGAGTTGGCCACTCCCTC
TCTGCGCGCTCGCTCGCTCACTGAGGCCGGGCGACCAAAGGTCGCCCGACGCCCGGGCTTTGCCCGGGCGGCCT
CAGTGAGCGAGCGAGCGCGCAGCTGCCTGCAGG

pAAV-NIRgal-R

BphP1-VP16-T2A-NLS(hnRNP_D)-GAL4-QPAS1

Protein sequence

MVAGHASGSPAFGTADLSNCEREEIHLAGSIQPHGALLVVSEPDHRIIQASANAAE
FLNLGSVLGVPLAEIDGDLLIKILPHLDPTAEGMPVAVRCRIGNPSTEYDGLMHRPP
EGGLIIELERAGPPIDLSGTLAPALERIRTAGSLRALCDDTALLFQQCTGYDRVMVYR
FDEQGHGEVFSERHVPGLESYFGNRYPSSDIPQMARRLYERQRVRVLVDVSYQPV
PLEPRLSPLTGRDLDMSGCFLRSMSPIHLQYLKNMGVRATLVVSLVVGGKLWGLV
ACHHYLPRFIHFELRAICELLAEAIATRITALESFAQSQSELFVQRLEQRMIEAITREGD
WRAAIFDTSQSILQPLHADGCALVYEDQIRTIGDVPSTQDVREIAGWLDRQPRAAV
TSTASLGLDVPELAHLTRMASGVVAAPISDHRGEFLMWFRPERVHTVTWGGDPKK
PFTMGDTPADLSPRRSFAKWHQVVEGTSDPWTAADLAAARTIGQTVADIVLQFR
AVRTLIAREQYEQFSSQVHASMQPVLITDAEGRILLMNDSFRDMLPAGSPSAVHLD
DLAGFFVESNDFLRNVAELIDHGRGWRGEVLLRGAGNRPLPLAVRADPVTRTEDQ
SLGFVLIFSDATDRRTADAARTRFQEGILASARPGVRLDSKSDLLHEKLLSALVENAQ
LAALEITYGVETGRIAELLEGVRQSMLRTAEVLGHLVQHAARTAGSDSSSNGSQNK
KEFDSAGSAGSAGTRAYSRARTKNNYGSTIEGLLDLPDDDAPEEAGLAAPRLSFLPA
GHTRRLSTAPPTDVSLGDELHLDGEDVAMAHADALDDFDLDMLGDGDSPGPGFT
PHDSAPYGALDMADFEFEQMFTDALGIDEYGGSGREGRGSLLTCGDVEENPGPH
MVSGYGKVSRRGGHQNSYKPYRSGGGGSKLLSSIEQACDICRLKKLKCSKEKPKCA
KCLKNNWECRYSPKTKRSPLTRAHLTEVESRLERLEQLFLLIFPREDLDMILKMDSLQ
DIKALLTGLFVQDNVNKDAVTDRLASVETDMPLTLRQHRISATSSSEESSNKGQRQ
LTVSPEFGKNMQAVTELHSRLIAAQQAMERDYWRLRELETRYRLVFDAAADAVMI
VSAGDMRIVEANRAAVNAISRVERGNDDLAGRDFLAEVAAADRDAVRDMLAQV
RQRGTALSVLVHLGRYDRAWMLRGSLMSSERRQVFLLHFTPVTTTPAIDDDDKGV
VASAADGAEGASDDAED*

pAAV-NIRgal-R

ITR-CamKII-BphP1-VP16-T2A-NLS(hnRNP_D)-GAL4-QPAS1-WPRE3-bGHpA-ITR

DNA sequence

CTGCGCGCTCGCTCGCTCACTGAGGCCGCCCGGGCAAAGCCCGGGCGTCGGGCGACCTTTGGTCGCCCGGCCTC
AGTGAGCGAGCGAGCGCGCAGAGAGGGAGTGGCCAACTCCATCACTAGGGGTTCCTGCGGCCGCAGATGCTGC
ACACTTGTGGACTAAGTTTGTTCGCATCCCCTTCTCCAACCCCCTCAGTACATCACCCTGGGGGAACAGGGTCCAC
TTGCTCCTGGGCCCACACAGTCCTGCAGTATTGTGTATATAAGGCCAGGGCAAAGAGGAGCAGGTTTTAAAGTGA
AAGGCAGGCAGGTGTTGGGGAGGCAGTTACCGGGGCAACGGGAACAGGGCGTTTCGGAGGTGGTTGCCATGGG
GACCTGGATGCTGACGAAGGCTCGCGAGGCTGTGAGCAGCCACAGTGCCCTGCTCAGAAGCCCCAAGCTCGTCA
GTCAAGCCGGTTCTCCGTTTGCACTCAGGAGCACGGGCAGGCGAGTGGCCCCTAGTTCTGGGGGCAGCGTGAAA
AGACGGTATCGATAAGCTTGATTCGAGGTACCTACCATGGTGGCAGGTCATGCCTCTGGCAGCCCCGCATTCGGG
ACCGCCGATCTTTCGAATTGCGAACGTGAAGAGATCCACCTCGCCGGCTCGATCCAGCCGCATGGCGCGCTTCTG
GTCGTCAGCGAGCCGGATCATCGCATCATCCAGGCCAGCGCCAACGCCGCGGAATTTCTGAATCTCGGAAGCGT
GCTCGGCGTTCCGCTCGCCGAGATCGACGGCGATCTGTTGATCAAGATCCTGCCGCATCTCGATCCCACCGCCGA
AGGCATGCCGGTCGCGGTGCGCTGCCGGATCGGCAATCCCTCCACGGAGTACGACGGTCTGATGCATCGGCCTC
CGGAAGGCGGGCTGATCATCGAGCTCGAACGTGCCGGCCCGCCGATCGATCTGTCCGGCACGCTGGCGCCGGC
GCTGGAGCGGATCCGCACGGCGGGCTCGCTGCGCGCGCTGTGCGATGACACCGCGCTGCTGTTTCAGCAGTGCA
CCGGCTACGACCGGGTGATGGTGTATCGCTTCGACGAGCAGGGCCACGGCGAAGTGTTCTCCGAGCGCCACGTG
CCCGGGCTCGAATCCTATTTCGGCAACCGCTATCCGTCGTCGGACATTCCGCAGATGGCGCGGCGGCTGTACGA
GCGGCAGCGCGTCCGCGTGCTGGTCGACGTCAGCTATCAGCCGGTGCCGCTGGAGCCGCGGCTGTCGCCGCTGA
CCGGGCGCGATCTCGACATGTCGGGCTGCTTCCTGCGCTCGATGTCGCCGATCCATCTGCAGTACCTGAAGAACA
TGGGCGTGCGCGCCACCCTGGTGGTGTCGCTGGTGGTCGGCGGCAAGCTGTGGGGCCTGGTTGCCTGTCACCAT
TATCTGCCGCGCTTCATCCATTTCGAGCTGCGGGCGATCTGCGAACTGCTCGCCGAAGCGATCGCGACGCGGATC
ACCGCGCTTGAGAGCTTCGCGCAGAGCCAGTCGGAGCTGTTCGTGCAGCGGCTCGAACAGCGCATGATCGAAGC
GATCACCCGTGAAGGCGATTGGCGCGCAGCGATTTTCGACACCAGCCAATCGATCCTGCAGCCGCTGCACGCCG
ACGGTTGCGCGCTGGTGTACGAAGACCAGATCAGGACCATCGGTGACGTACCTTCCACGCAGGATGTTCGCGAG
ATCGCCGGGTGGCTCGATCGCCAGCCACGTGCGGCGGTGACCTCGACCGCGTCGCTCGGTCTCGACGTGCCGGA
GCTCGCGCATCTGACGCGGATGGCGAGCGGCGTGGTCGCGGCGCCGATTTCGGATCATCGCGGCGAGTTTCTGA
TGTGGTTCCGCCCCGAGCGCGTCCACACCGTTACCTGGGGCGGCGATCCGAAGAAGCCGTTCACGATGGGCGAT
ACACCGGCGGATCTGTCGCCGCGGCGCTCCTTCGCCAAATGGCATCAGGTTGTCGAAGGCACGTCCGATCCGTG
GACGGCCGCCGATCTCGCCGCGGCTCGCACCATCGGTCAGACCGTCGCCGACATCGTGCTGCAATTCCGCGCGG
TGCGGACACTGATCGCCCGCGAACAGTACGAACAGTTTTCGTCCCAGGTGCACGCTTCGATGCAGCCGGTGCTG
ATCACCGACGCCGAAGGCCGCATCCTGCTGATGAACGACTCGTTCCGCGACATGTTGCCGGCGGGGTCGCCATC
CGCCGTCCATCTCGACGATCTCGCCGGGTTCTTCGTCGAATCGAACGATTTCCTGCGCAACGTCGCCGAACTGAT
CGATCACGGCCGCGGGTGGCGCGGCGAAGTTCTGCTGCGCGGCGCAGGTAATCGCCCGTTGCCGCTGGCAGTG
CGCGCCGATCCGGTGACGCGCACGGAGGACCAGTCGCTCGGCTTCGTGCTGATCTTCAGCGACGCTACCGATCG
TCGCACCGCAGATGCCGCACGCACGCGTTTCCAGGAAGGCATTCTTGCCAGCGCACGTCCCGGCGTGCGGCTCG
ACTCCAAGTCCGACCTCTTGCACGAGAAGCTGCTGTCCGCGCTGGTCGAGAACGCGCAGCTTGCCGCATTGGAA
ATTACTTACGGCGTCGAGACCGGACGCATCGCCGAGCTGCTCGAAGGCGTTCGCCAGTCGATGCTGCGCACCGC
CGAAGTGCTCGGCCATCTGGTGCAGCACGCGGCGCGCACGGCCGGCAGCGACAGCTCGAGCAATGGCTCGCAG

AACAAGAAGGAATTCGATAGTGCTGGTAGTGCTGGTAGTGCTGGTACTAGAGCGTACAGCCGCGCGCGTACGAA
AAACAATTACGGGTCTACCATCGAGGGCCTGCTCGATCTCCCGGACGACGACGCCCCCGAAGAGGCGGGGCTG
GCGGCTCCGCGCCTGTCCTTTCTCCCCGCGGACACACGCGCAGACTGTCGACGGCCCCCCGACCGATGTCAG
CCTGGGGGACGAGCTCCACTTAGACGGCGAGGACGTGGCGATGGCGCATGCCGACGCGCTAGACGATTTCGAT
CTGGACATGTTGGGGGACGGGGATTCCCCGGGTCCGGGATTTACCCCCCACGACTCCGCCCCCTACGGCGCTCT
GGATATGGCCGACTTCGAGTTTGAGCAGATGTTTACCGATGCCCTTGGAATTGACGAGTACGGTGGGAGCGGCC
GCGAGGGCAGAGGAAGTCTGCTAACATGCGGTGACGTCGAGGAGAATCCTGGCCCACATATGGTGAGTGGCTAT
GGGAAAGTATCCCGACGGGGAGGCCATCAGAATAGTTACAAACCATACAGATCTGGAGGTGGTGGAAGCAAGC
TACTGTCTTCTATCGAACAAGCATGCGATATTTGCCGACTTAAAAAGCTCAAGTGCTCCAAAGAAAAACCGAAGT
GCGCCAAGTGTCTGAAGAACAACTGGGAGTGTCGCTACTCTCCCAAAACCAAAAGGTCTCCGCTGACTAGGGCA
CATCTGACAGAAGTGGAATCAAGGCTAGAAAGACTGGAACAGCTATTTCTACTGATTTTTCCTCGAGAAGACCTT
GACATGATTTTGAAAATGGATTCTTTACAGGATATAAAAGCATTGTTAACAGGATTATTTGTACAAGATAATGTGA
ATAAAGATGCCGTCACAGATAGATTGGCTTCAGTGGAGACTGATATGCCTCTAACATTGAGACAGCATAGAATAA
GTGCGACATCATCATCGGAAGAGAGTAGTAACAAAGGTCAAAGACAGTTGACTGTATCGCCGGAATTCGGCAAG
AACATGCAGGCGGTCACCGAGCTGCATTCCCGGCTGATCGCTGCGCAGCAGGCGATGGAGCGCGACTATTGGCG
GTTGCGTGAATTGGAGACTCGCTACCGCCTGGTGTTCGACGCTGCCGCCGATGCGGTGATGATCGTCTCCGCCGG
CGACATGCGCATCGTCGAAGCCAACCGGGCGGCGGTGAATGCGATCAGCCGCGTCGAGCGCGGCAATGACGAC
CTTGCGGGGCGTGATTTCCTCGCCGAAGTGGCGGCTGCCGATCGCGATGCGGTGCGCGACATGCTGGCCCAGGT
GCGTCAGCGCGGCACCGCACTCAGCGTCCTCGTTCATCTCGGCCGTTACGACCGCGCCTGGATGCTGCGCGGTTC
GCTGATGTCGTCCGAGCGTCGTCAGGTTTTCCTGCTGCACTTCACCCCGGTGACCACGACTCCCGCGATCGACGA
CGACGACAAAGGTGTCGTTGCTTCTGCTGCCGACGGTGCAGAGGGCGCCTCAGACGACGCAGAGGATTAAAGCC
CTAGGCGCTCGAGATAATCAACCTCTGGATTACAAAATTTGTGAAAGATTGACTGGTATTCTTAACTATGTTGCTC
CTTTTACGCTATGTGGATACGCTGCTTTAATGCCTTTGTATCATGCTATTGCTTCCCGTATGGCTTTCATTTTCTCCTC
CTTGTATAAATCCTGGTTAGTTCTTGCCACGGCGGAACTCATCGCCGCCTGCCTTGCCCGCTGCTGGACAGGGGC
TCGGCTGTTGGGCACTGACAATTCCGTGGTGTTTATTTGTGAAATTTGTGATGCTATTGCTTTATTTGTAACCATCTA
GCTTTATTTGTGAAATTTGTGATGCTATTGCTTTATTTGTAACCATTATAAGCTGCAATAAACAAGTTAACAACAAC
AATTGCATTCATTTTATGTTTCAGGTTCAGGGGGAGATGTGGGAGGTTTTTTAAAGCGGCCGCAGGAACCCCTAGT
GATGGAGTTGGCCACTCCCTCTCTGCGCGCTCGCTCGCTCACTGAGGCCGGGCGACCAAAGGTCGCCCGACGCC
CGGGCTTTGCCCGGGCGGCCTCAGTGAGCGAGCGAGCGCGCAGCTGCCTGCAGG

pAAV-U5-Gluc

ITR-U5-Gluc-WPRE3-bGHpA-ITR

DNA sequence

CCTGCAGGCAGCTGCGCGCTCGCTCGCTCACTGAGGCCGCCCGGGCGTCGGGCGACCTTTGGTCGCCCGGCCTC
AGTGAGCGAGCGAGCGCGCAGAGAGGGAGTGGCCAACTCCATCACTAGGGGTTCCTGCGGCCGCACGCGTGGC
GTTTTGCGCTGCTTCGCGAATATTAAGGTACGGGAGGTACTTGGAGCGGCCGCAATAAAATATCTTTATTTTCATT
ACATCTGTGTGTTGGTTTTTTGTGTGAATCGATAGTACTAACATACGCTCTCCATCAAAACAAAACGAAACAAAAC
AAACTAGCAAAATAGGCTGTCCCCAGTGCAAGTGCAGGTGCCAGAACATTTCTCTATCGATAGGTACCGAGTTTC
TAGACGGAGTACTGTCCTCCGAGCGGAGTACTGTCCTCCGACTCGAGCGGAGTACTGTCCTCCGATCGGAGTACT
GTCCTCCGCGAATTCCGGAGTACTGTCCTCCGAAGACGCTAGCGGGGGGCTATAAAAGGGGGTGGGGGCGTTCG
TCCTCACTCTAGATCTGCGATCTAAGTAAGCTTGGCATTCCGGTACTGTTGGTAAAGCCGGACGTCCTCTAGCCAC
CGCCACCATGGGAGTCAAAGTTCTGTTTGCCCTGATCTGCATCGCTGTGGCCGAGGCCAAGCCCACCGAGAACA
ACGAAGACTTCAACATCGTGGCCGTGGCCAGCAACTTCGCGACCACGGATCTCGATGCTGACCGCGGGAAGTTG
CCCGGCAAGAAGCTGCCGCTGGAGGTGCTCAAAGAGATGGAAGCCAATGCCCGGAAAGCTGGCTGCACCAGGG
GCTGTCTGATCTGCCTGTCCCACATCAAGTGCACGCCCAAGATGAAGAAGTTCATCCCAGGACGCTGCCACACCT
ACGAAGGCGACAAAGAGTCCGCACAGGGCGGCATAGGCGAGGCGATCGTCGACATTCCTGAGATTCCTGGGTT
CAAGGACTTGGAGCCCATGGAGCAGTTCATCGCACAGGTCGATCTGTGTGTGGACTGCACAACTGGCTGCCTCA
AAGGGCTTGCCAACGTGCAGTGTTCTGACCTGCTCAAGAAGTGGCTGCCGCAACGCTGTGCGACCTTTGCCAGCA
AGATCCAGGGCCAGGTGGACAAGATCAAGGGGGCCGGTGGTGACTAAGGCGCGCCTCAACCTCTGGATTACAA
AATTTGTGAAAGATTGACTGGTATTCTTAACTATGTTGCTCCTTTTACGCTATGTGGATACGCTGCTTTAATGCCTTT
GTATCATGCTATTGCTTCCCGTATGGCTTTCATTTTCTCCTCCTTGTATAAATCCTGGTTAGTTCTTGCCACGGCGGA
ACTCATCGCCGCCTGCCTTGCCCGCTGCTGGACAGGGGCTCGGCTGTTGGGCACTGACAATTCCGTGGTGTTTAT
TTGTGAAATTTGTGATGCTATTGCTTTATTTGTAACCATCTAGCTTTATTTGTGAAATTTGTGATGCTATTGCTTTATT
TGTAACCATTATAAGCTGCAATAAACAAGTTAACAACAACAATTGCATTCATTTTATGTTTCAGGTTCAGGGGGAG
ATGTGGGAGGTTTTTTAAAGCGGCCGCAGGAACCCCTAGTGATGGAGTTGGCCACTCCCTCTCTGCGCGCTCGCT
CGCTCACTGAGGCCGGGCGACCAAAGGTCGCCCGACGCCCGGGCTTTGCCCGGGCGGCCTCAGTGAGCGAGCG
AGCGCGCAGCTGCCTGCAGG
